# Scope and Limitations of γ-Valerolactone (GVL) as a Green Solvent to be Used with Base for Fmoc Removal in Solid Phase Peptide Synthesis

**DOI:** 10.3390/molecules24214004

**Published:** 2019-11-05

**Authors:** Ashish Kumar, Anamika Sharma, Beatriz G. de la Torre, Fernando Albericio

**Affiliations:** 1 Peptide Science Laboratory, School of Chemistry and Physics, University of KwaZulu-Natal, Durban 4001, South Africa; ashishpy12@gmail.com (A.K.); anamika.aug14@gmail.com (A.S.); 2 KwaZulu-Natal Research Innovation and Sequencing Platform (KRISP), School of Laboratory Medicine and Medical Sciences, College of Health Sciences, University of KwaZulu-Natal, Durban 4041, South Africa; 3 Department of Organic Chemistry, University of Barcelona, Martí i Franqués 1-11, 08028 Barcelona, Spain; 4 CIBER-BBN, Networking Centre on Bioengineering, Biomaterials and Nanomedicine, Barcelona Science Park, Baldiri Reixac 10, 08028 Barcelona, Spain

**Keywords:** piperidine, 4-methylpiperidine, acyl piperidide, NMR, IR, DFT

## Abstract

GVL is a green solvent used in Fmoc-based solid-phase peptide synthesis. It is susceptible to ring opening in the presence of bases such as piperidines, which are used to remove the Fmoc protecting group. Here we studied the formation of the corresponding acyl piperidides by time-dependent monitoring using NMR. The results, corroborated by theoretical calculations, indicate that a solution of piperidines in GVL should be prepared daily for a better Fmoc removal.

## 1. Introduction

In the pharmaceutical industry and synthetic chemical manufacturing sector, solvents account for 80–90% of the total waste [1,2]. The increasing demand for synthetic peptides in the pharmaceutical market calls for attempts to tackle greening the production of these compounds. In this regard, recent years have witnessed an increase in the size and complexity of the peptides required for both research and production purposes [3]. Small peptides can be synthesized in solution phase; however, this approach is not appropriate for the preparation of medium-sized and long peptides because it would require many synthetic and purification steps [4,5,6,7,8]. In this regard, solid-phase peptide synthesis (SPPS), which was introduced by Merrifield [9] and later fine-tuned by Carpino et al. [10] by the introduction of the fluorenylmethoxycarbonyl (Fmoc) group, was a milestone in the field. In SPPS, the carboxyl group of the C-terminal amino acid is permanently protected through its attachment to a solid support, whereas the temporary *N*^α^ protecting group is subsequently removed to allow the repetitive incorporation of the rest of the residues until completion of the peptide [11]. The excess of reagents and byproducts generated during the synthesis can be easily removed by filtration and several washings, the latter requiring excess amounts of solvent [11]. DMF is the solvent most widely used in SPPS, but it has been classified as a reprotoxic solvent and a substance of very high concern (SVHC) [12,13]. Several attempts have been made to replace DMF with a green solvent, according to the solvent selection guide [14]. Our group and others have proposed the use of less hazardous and green solvents such as water [15], acetonitrile (ACN), tetrahydrofuran (THF) [16], 2-methyltetrahydrofuran (2-MeTHF) [17,18], *N*-formylmorpholine (NFM) [19], γ-valerolactone (GVL) [12,19,20], *N*-butylpyrrolidone (NBP) [21], and propylene carbonate [22], and even mixtures of green solvents [23]. In order to be used in SPPS, solvents must have the capacity to swell the resins to allow the reagents to reach the active functional groups, and they must also be efficient during the coupling and deprotection steps, both of which are crucial [11]. GVL is obtained from renewable lignocellulosic biomass and it is therefore non-toxic and biodegradable [24]. In this regard, we recently synthesized medium and large difficult peptides using GVL as solvent in all steps and achieved results comparable to those of DMF. However, we identified that a capped peptide forms through the acylation of Gly with 4-hydroxypentanoic acid during the removal of Fmoc from Fmoc-Gly using piperidine, which is the less least hindered amino acid. This side reaction can be overcome by introducing the Gly in the form of a Fmoc-dipeptide with the subsequent amino acid as demonstrated for the preparation of ABRF 1992 (H-GVRGDKGNP GWPGAPY-NH_2_) a difficult peptide consisting of five Gly residues using a microwave (MW)-assisted peptide synthesizer [20]. The reaction of the *N*^α^-amino function of the Gly residue with GVL was catalyzed by the piperidine (PIP) used to remove the Fmoc group. At this point, we were aware of GVL ring opening with base reported by Chalid et al. [25]. In the present study, we examined the ring opening of this green solvent in the presence of PIP and 4-methylpiperidine (4-MP), which are the most common bases used to remove the Fmoc group. 4-MP is considered superior to PIP [26]. The great disadvantage of the latter is its current legal status as a controlled substance regulated by the Drug Enforcement Agency. This classification is due to the fact that it is used in the synthesis of psychotropic drugs and thus special permission is required for its purchase in some countries [27]. 4-MP, on the other hand, is an effective substitute for Fmoc removal in SPPS and it costs less than PIP [27]. The final structures of the product formed were further established with the help of NMR (^1^H, ^13^C and 2D including HSQC, HMBC, COSY) and IR. Theoretical calculations were also performed to better understand the reaction pathway that led to the ring opening of GVL in presence of base. Transition states (TS) were calculated, and the reaction pathway was confirmed by intrinsic reaction coordinate (IRC) calculations.

## 2. Results and Discussion

**Scheme 1:**
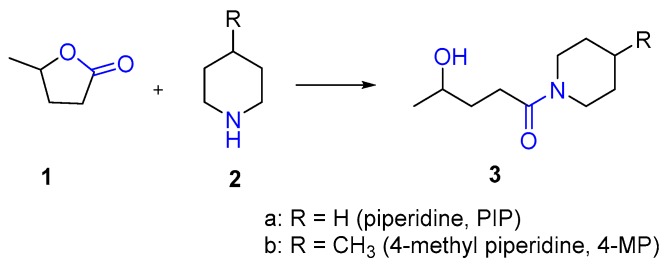
Ring opening of GVL in the presence of PIP and 4-MP.

In our earlier work, we demonstrated that GVL (1) and DMF show a similar performance for the SPPS of small, medium and also long model peptides in a microwave-assisted automated peptide synthesizer [13]. The physical properties of GVL, such as melting point, boiling point and viscosity, were acceptable for both manual SPPS and using peptide synthesizers (Table 1) [13]. However, our main concern was the instability of GVL (ring opening) with PIP (2a) and 4-MP (2b) and its potential impact on the storage of PIP/4-MP solutions in GVL (1) during the synthetic process (Scheme 1).

**Figure 1:**
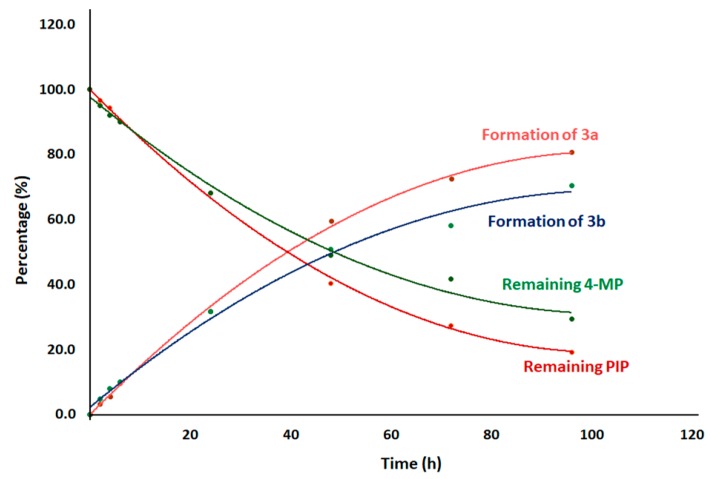
Evolution of the reaction of piperidines with GVL.

**Figure 2:**
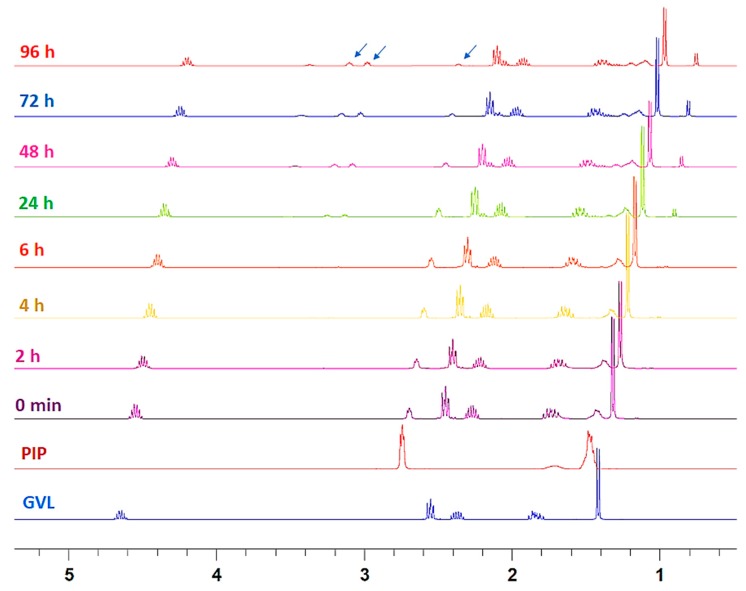
Time-dependent ^1^H-NMR study of the formation of piperidide **3a** by ring opening of GVL with 20% PIP. Arrows indicate the signals selected for calculation of the percentage of ring opening.

**Figure 3:**
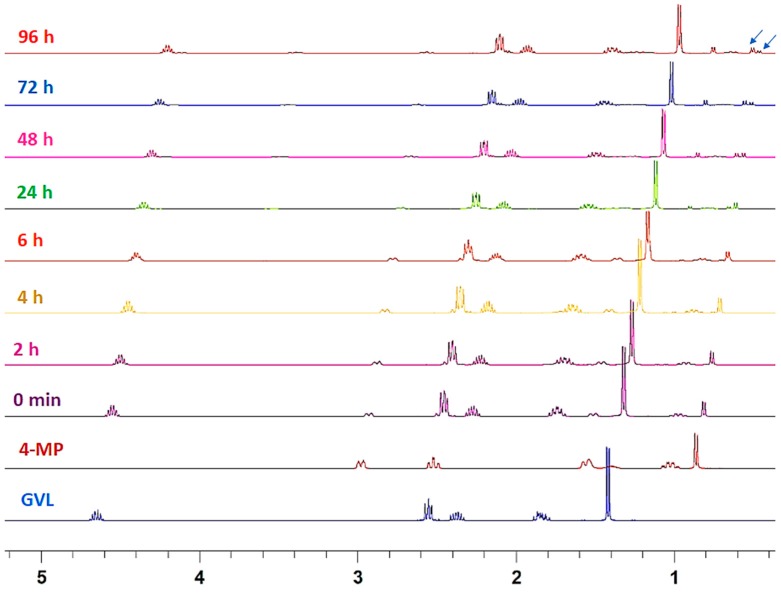
Time-dependent ^1^H-NMR study of the formation of piperidide **3b** by ring opening of GVL with 20% 4-MP. Arrows indicate the signals selected for calculation of the percentage of ring opening.

In this regard, the ring opening of GVL was studied in the presence of 20% PIP (2.02 M) and 4-MP (1.69 M), considered as standard solutions for Fmoc removal in SPPS (Figure 1). Both solutions were left at rt for 96 h and analyzed by ^1^H-NMR (Figure 2 and Figure 3) at 0 min, 2 h, 4 h, 6 h, 24 h, 48 h, 72 h, and 96 h (Individual NMR details can be found in Supplementary Information). We detected a new set of signals that were not related to GVL or to PIP alone. These new signals showed a steady increase over time, accompanied by a simultaneous decrease of the GVL and PIP signals. Further analysis of the new signals in ^1^H-NMR led us to conclude that they corresponded to the formation of 4-hydroxypentanoic piperidide (**3a**) and 4-hydroxypentanoic 4-methylpiperidide (**3b**) in the case of PIP and 4-MP, respectively. Figure 1 shows the evolution of the formation of these two piperidides **3a** and **3b**.

The percentage of **3a** formation was calculated based on the conversion of α-CH_2_ of PIP (2.79 ppm) into new signals at 3.42 and 3.52 ppm (as shown by arrow in Figure 2). For 4-MP, CH_3_ at δ position of 4-MP appeared as a doublet at 0.91 ppm. Upon reaction with GVL, 4-MP gave rise to a new signal at 0.96 ppm, indicating the formation of **3b** (as shown by arrow in Figure 3). Overall, after 24 h, two thirds of the piperidines remained in the solution. At 96 h, this percentage had decreased to 20% for PIP and 36% for 4-MP.

**Figure 4:**
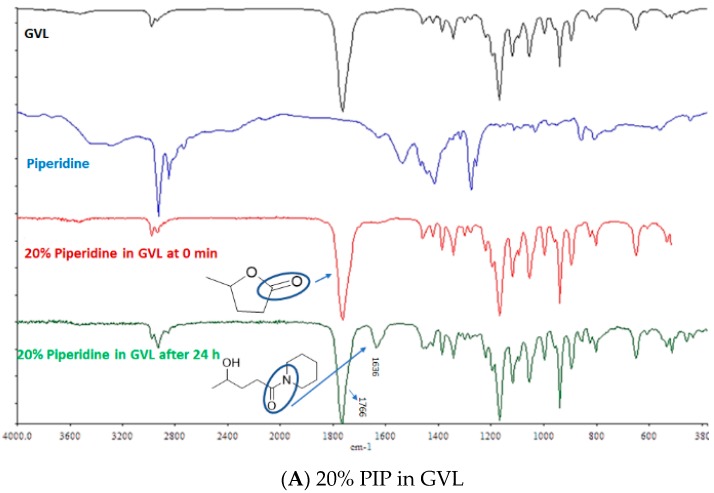

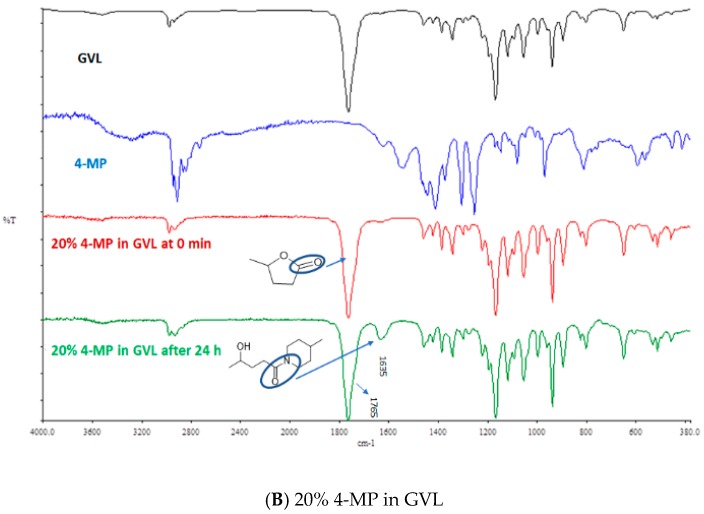
FT-IR comparison of base with GVL: (**A**) 20% PIP in GVL and (**B**) 20% 4-MP in GVL.

The ring opening of GVL in the presence of base (**3a** and **3b**) was also confirmed by FT-IR (Figure 4). As shown in the FT-IR of GVL, a strong peak was observed with a stretching frequency of 1766 cm^−1^, which corresponds to lactones (cyclic ester: C=O bond). However, with time, and in the presence of 20% base (PIP and 4-MP) in GVL another peak at frequency 1636 cm^−1^ was observed, which corresponds to the amide bond, thereby further confirming the formation of the amide bond as a result of ring opening [25].

**Figure 5:**
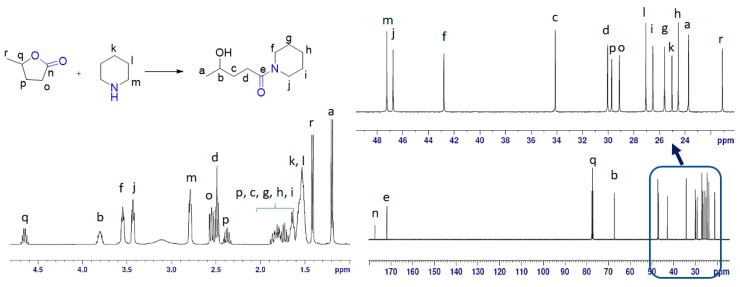
NMR study of equimolar solution of GVL and PIP under MW at 90 °C for 1 h. Left part explains the ^1^H-NMR whereas the bottom right side of the figure shows the ^13^C-NMR with an expansion of the 10–50 ppm region in the top right corner.

**Figure 6:**
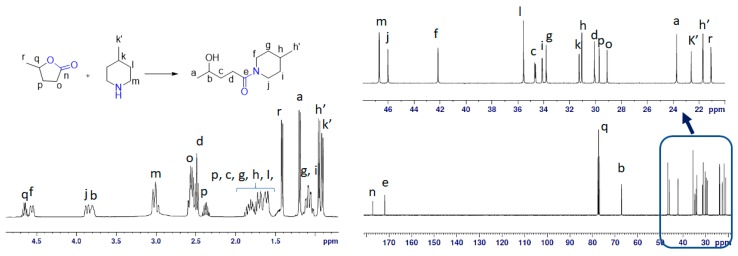
NMR study of an equimolar solution of GVL and 4-MP under MW at 90 °C for 1 h. The left part explains the ^1^H-NMR whereas bottom right side of the figure shows the ^13^C-NMR with an expansion of the 10–50 ppm region in the top right corner.

To confirm the results, in another experiment, an equimolar solution of PIP and 4-MP were left to react with GVL separately under microwave conditions (90 °C) for 1 h, as this condition is quite strong for peptide synthesis [12]. After the reaction, NMR was performed (Figure 5 and Figure 6) and the percentage of **3a** and **3b** formation was calculated as explained above.

**Figure 7:**
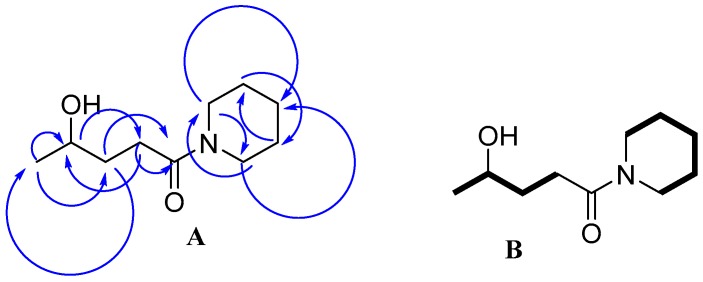
(**A**) Important HMBC correlations; (**B**) COSY correlations (Bold line).

The ^1^H chemical shift in the spectra was the mixture of GVL, PIP and **3a,** as shown in Figure 5**.** The ^13^C-NMR spectrum revealed the presence of 18 signals. These consisted of two carbonyl (CO), two methine (CH), 12 methylene (CH_2_), and two methyl (CH_3_) groups, which were related to the 10 signals for **3a**, five signals for GVL and three signals for PIP (Figure 5). The ^13^C-NMR values for all carbon signals were assigned on the basis of HSQC and HMBC spectral data. The suggested structure of **3a** was supported by key HMBC correlations: H-a/C-b, C-c; H-b/C-d; H-c/C-a, C-e; H-d/C-b, C-c, C-e; H-f/ C-e, C-h, C-j; H-g/C-f, C-i; H-h/C-f, C-j; H-i/C-g, C-j; H-j/C-e, C-f, C-h (Figure 7A). The structure was further supported by the COSY spectrum (H-a/H-b; H-b/H-c; H-c/H-d; H-f/H-g; H-g/H-h; H-h/H-i; H-i/ H-j) (Figure 7B). From the ^1^H-NMR, 62% of **3a** was detected in the case of PIP. However, in case of 4-MP, only 57% of **3b** was obtained, thereby indicating the slightly higher stability of 4-MP in GVL compared to that of PIP.

A theoretical investigation has been performed to understand the mechanism of ring opening of GVL in presence of PIP and 4-MP. Geometry optimization was performed using Gaussian09 program package, employing the B3LYP (Becke three parameters Lee–Yang–Parr exchange correlation functional) and the 6-311G++(d,p) as basis set. Geometries were optimized, and the frequency calculations showed no negative eigen value [28]. The thermodynamics parameters for PIP and 4-MP with GVL are given in Table 2.

**Figure 8:**
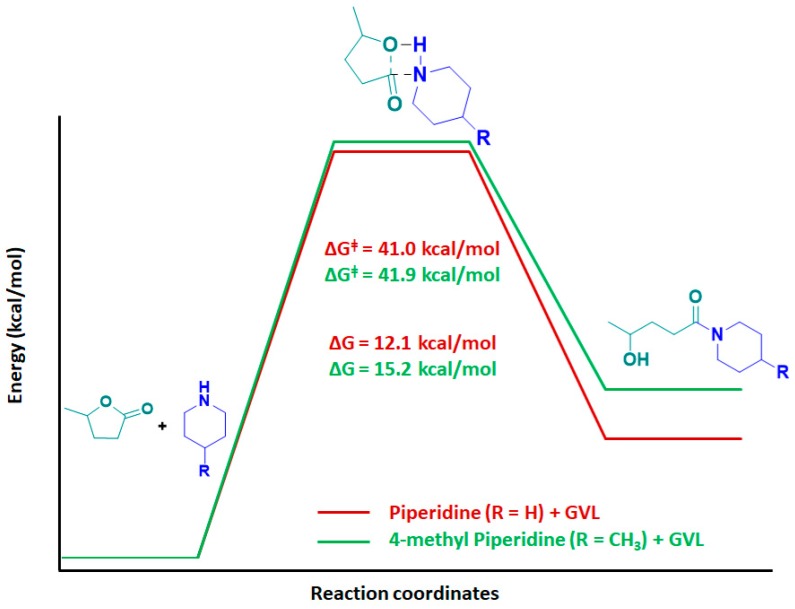
Reaction pathway to explain GVL ring opening in the presence of PIP and 4-MP.

According to the enthalpy values (ΔH), from a thermodynamic perspective, the reaction was slightly exothermic. However, Gibb’s free energy (ΔG) confirmed the non-spontaneity of the reaction. In order to explain the mechanism, a four-membered TS was calculated between GVL and PIP /4-MP (Figure 8). IRC calculations were performed to confirm the path of the reaction via the calculated TS [29]. The activation energy for PIP and 4-MP was 41.9 kcal/mol and 41.0 kcal/mol, respectively.

**Figure 9:**
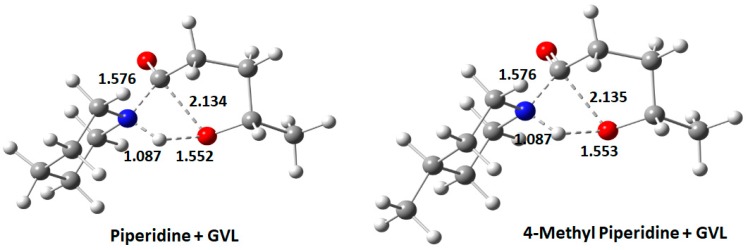
Transition state for PIP + GVL and 4-MP + GVL.

The TSs of the two cases looked identical with almost similar bond lengths (Figure 9). In the case of bases, the usual length of the NH bond was 1.02 Å, which increased slightly to 1.09 Å in TS. Even the C–O bond length in GVL increased from 1.36 Å to 2.14 Å. The reaction proceeded with the breaking of the C–O bond in GVL, causing an increase in bond length from 1.36 Å to 1.89 Å, followed by slight elongation of N–H bond from 1.02 Å to 1.04 Å until the TS was obtained, as shown in Figure 8. From Figure 8 and Table 2, it can be appreciated that 4-MP required slightly more activation energy to attain the TS compared to PIP.

## 3. Materials and Methods

### 3.1. General Information

All reagents and solvents were obtained from commercial suppliers. PIP, 4-MP, GVL and CDCl_3_ (with TMS as internal standard) were purchased from Sigma-Aldrich (St Louis, MO, USA). The ^1^H-NMR and ^13^C-NMR spectra were recorded on an Avance III 400 MHz NMR spectrometer (Bruker, Billerica, MA, USA). The spectral width was 20.0209 ppm and 238.8430 ppm for ^1^H-NMR and ^13^C-NMR, respectively, the number of scans were 16 and 1024 for ^1^H-NMR and ^13^C-NMR, respectively, and number of dummy scans (DS) was 2 and 4 in ^1^H-NMR and ^13^C-NMR, respectively. The pulse delay was 1 sec and 2 sec for ^1^H-NMR and ^13^C-NMR, respectively. Infrared spectra were recorded on a Spectrum 100 FT-IR spectrometer (Perkin Elmer, Shelton, CT, USA).

### 3.2. Procedure

One mL of 20% PIP and 4-MP in a solution of GVL (*v*/*v*) was prepared separately at rt. Next, 40 µL of sample was withdrawn from the reaction mixture and 400 µL of CDCl_3_ was added. The resulting mixture was analyzed by ^1^H-NMR at 0 min, 2 h, 4 h, 6 h, 24 h, 48 h, 72 h, and 96 h. The percentage of GVL ring opening was calculated by integration of the α-CH_2_ signal of PIP and that of **3a,** and the δ-CH_3_ signal of 4-MP and that of **3b,** respectively. Equimolar solutions (1 equiv. each) of GVL with PIP and GVL with 4-MP were prepared and subjected to microwave (MW) conditions at 90 °C for 1 h, followed by NMR analysis. IR was recorded for GVL, PIP and 4-MP at 0 min and 24 h, as well as for 20% PIP and 4-MP in GVL (*v*/*v*).

### 3.3. Theoretical Calculations

Geometry optimization was performed by DFT calculations using the Gaussian09 program package (Wallingford, CT, USA) [28], employing the B3LYP (Becke’s three-parameter Lee–Yang–Parr exchange correlation functional) and the 6-311G++(d,p) as basis set [28]. No solvent corrections were made with these calculations. No negative eigen value was observed for the frequency calculations. Transition state (TS) was calculated using the above basis set with one negative eigen value. TS were also confirmed by IRC in order to confirm the reaction path [29]. All calculations were performed in gaseous state at 298 K.

## 4. Conclusions

GVL undergoes ring opening with bases like PIP and 4-MP, which are commonly used for Fmoc removal in SPPS. The present study demonstrates that 4-MP in GVL shows slightly higher stability than PIP. These results were confirmed via the formation of a TS with an activation energy of 41.0 and 41.9 kcal/mol for PIP and 4-MP, respectively. These findings indicate that a solution of either PIP, or preferably 4-MP, in GVL should be prepared daily in order to ensure optimal performance of the Fmoc removal solutions. In addition, initial preparation of a solution of 25% base in GVL could also be used.

## Figures and Tables

**Table 1:** Physical properties of DMF vs. GVL.

	DMF	GVL
**Density (g/mL)**	0.95	1.05
**Viscosity (cP, 25** **°C)**	0.92	1.86
**Melting point (** **°C)**	−31.0	−31.0
**Boiling point (** **°C)**	153	207–208
**Flash point (** **°C)**	58	96

**Table 2:** Energetics for the ring opening of GVL in the presence of PIP and 4-MP.

	Base *	ΔH (kcal/mol)	ΔG^#^ (kcal/mol/K)	ΔS (cal/mol)	K_eq_
**Thermodynamics**	PIP	−0.2	12.1	−10.4	0.008
	4-MP	−0.1	15.2	−12.4	0.002
**Kinetics**	PIP	35.7	41.0	−17.9	-
	4-MP	35.8	41.9	−20.2	-

* Reaction with GVL; ^#^In case of kinetics ΔG refers to activation energy (ΔG≠).

## Supplementary Materials

The following are available online.
