# Supplementary material for: Scope and Limitations of γ-Valerolactone (GVL) as a Green Solvent to be Used with Base for Fmoc Removal in Solid Phase Peptide Synthesis
Source: Molecules. 2019 Nov 5;24(21):4004. doi: 10.3390/molecules24214004 (PMC6864637; doi:10.3390/molecules24214004)
Supplement: Supplementary file 1 [file molecules-24-04004-s001.pdf]

Supporting Information

Scope and Limitations of γ-Valerolactone (GVL) as a Green Solvent to be Used with Base for Fmoc Removal in Solid Phase Peptide Synthesis

Ashish Kumar [1,2], Anamika Sharma [1,2], Beatriz G. de la Torre [2,*] and Fernando Albericio [1,3,4,*]

[1] Peptide Science Laboratory, School of Chemistry and Physics, University of KwaZulu-Natal, Durban 4001, South Africa; ashishpy12@gmail.com (A.K.); anamika.aug14@gmail.com (A.S.)

[2] KwaZulu-Natal Research Innovation and Sequencing Platform (KRISP), School of Laboratory Medicine and Medical Sciences, College of Health Sciences, University of KwaZulu-Natal, Durban 4041, South Africa

[3] Department of Organic Chemistry, University of Barcelona, Martí i Franqués 1-11, Barcelona 08028, Spain

[4] CIBER-BBN, Networking Centre on Bioengineering, Biomaterials and Nanomedicine, Barcelona Science Park, Baldiri Reixac 10, Barcelona 08028, Spain

* Correspondence: garciadelatorreb@ukzn.ac.za (B.G.d.l.T.); Albericio@ukzn.ac.za (F.A.)

Sl. No. Content

1 : **Figure 1:** ^{1}H NMR of GVL

2 : **Figure 2:** ^{13}C NMR of GVL

3 : **Figure 3:** ^{1}H NMR of PIP

4 : **Figure 4:** ^{13}C NMR of PIP

5 : **Figure 5:** ^{1}H NMR of 4-MP

6 : **Figure 6:** ^{13}C NMR of 4-MP

7 : **Figure 7:** ^{1}H NMR of 20% PIP in GVL at 0 min

8 : **Figure 8:** ^{1}H NMR of 20% PIP in GVL after 2 h

9 : **Figure 9:** ^{1}H NMR of 20% PIP in GVL after 4 h

10 : **Figure 10:** ^{1}H NMR of 20% PIP in GVL after 6 h

11 : **Figure 11:** ^{1}H NMR of 20% PIP in GVL after 24 h

12 : **Figure 12:** ^{1}H NMR of 20% PIP in GVL after 48 h

13 : **Figure 13:** ^{1}H NMR of 20% PIP in GVL after 72 h

14 : **Figure 14:** ^{1}H NMR of 20% PIP in GVL after 96 h

15 : **Figure 15:** ^{1}H NMR of 20% 4-MP in GVL at 0 min

16 : **Figure 16:** ^{1}H NMR of 20% 4-MP in GVL after 2 h

17 : **Figure 17:** ^{1}H NMR of 20% 4-MP in GVL after 4 h

18 : **Figure 18:** ^{1}H NMR of 20% 4-MP in GVL after 6 h

19 : **Figure 19:** ^{1}H NMR of 20% 4-MP in GVL after 24 h

20 : **Figure 20:** ^{1}H NMR of 20% 4-MP in GVL after 48 h

21 : **Figure 21:** ^{1}H NMR of 20% 4-MP in GVL after 72 h

22 : **Figure 22:** ^{1}H NMR of 20% 4-MP in GVL after 96h

23 : **Figure 23:** ^{1}H NMR of equimolar solution of GVL and PIP after 1h MW at 90 ℃

24 : **Figure 24:** ^{13}C NMR of equimolar solution of GVL and PIP after 1h MW at 90 ℃

25 : **Figure 25:** DEPT 135 of equimolar solution of GVL and PIP after 1h MW at 90 ℃

26 : **Figure 26:** HSQC of equimolar solution of GVL and PIP after 1h MW at 90 ℃

27 : **Figure 27:** HMBC of equimolar solution of GVL and PIP after 1h MW at 90 ℃

28 : **Figure 28:** COSY of equimolar solution of GVL and PIP after 1h MW at 90 ℃

29 : **Figure 29:** ^{1}H NMR of equimolar solution of GVL and 4-MP after 1h MW at 90 ℃

30 : **Figure 30:** ^{13}C NMR of equimolar solution of GVL and 4-MP after 1h MW at 90 ℃

31 : **Table 1:** ^{1}H, ^{13}C NMR and HBMC correlation of mixture of Product (**3a)** GVL and PIP

32 : Internal Coordinates for GVL

33 : Internal Coordinates for PIP

34 : Internal Coordinates for 4-MP

35 : Internal Coordinates for TS for GVL and PIP

36 : Internal Coordinates for TS for GVL and 4-MP

37 : **Figure 31:** Optimized geometry of GVL, PIP and 4-MP with bond lengths

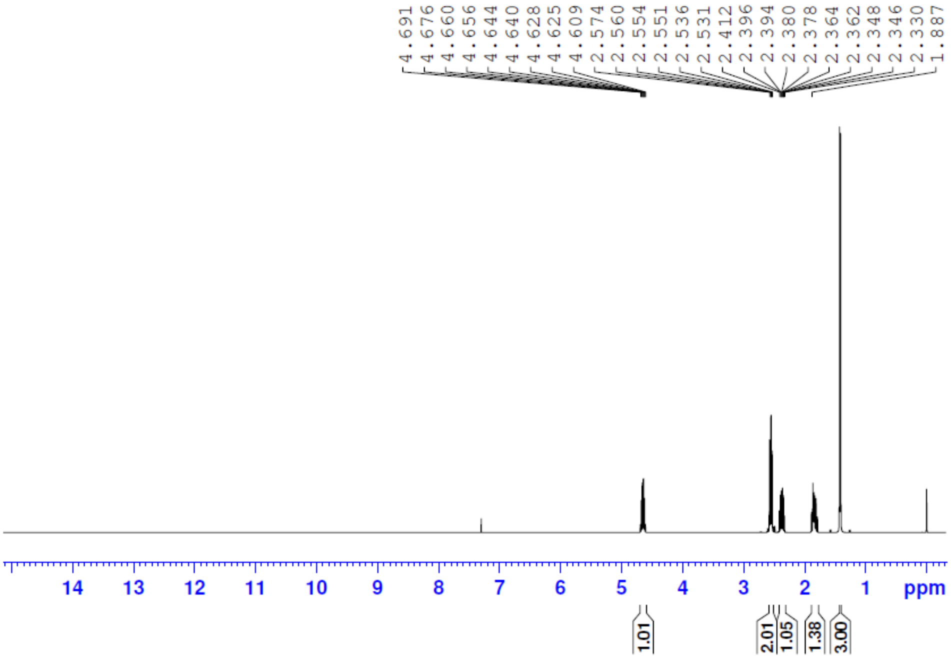

Figure 1.^{1}H NMR of GVL

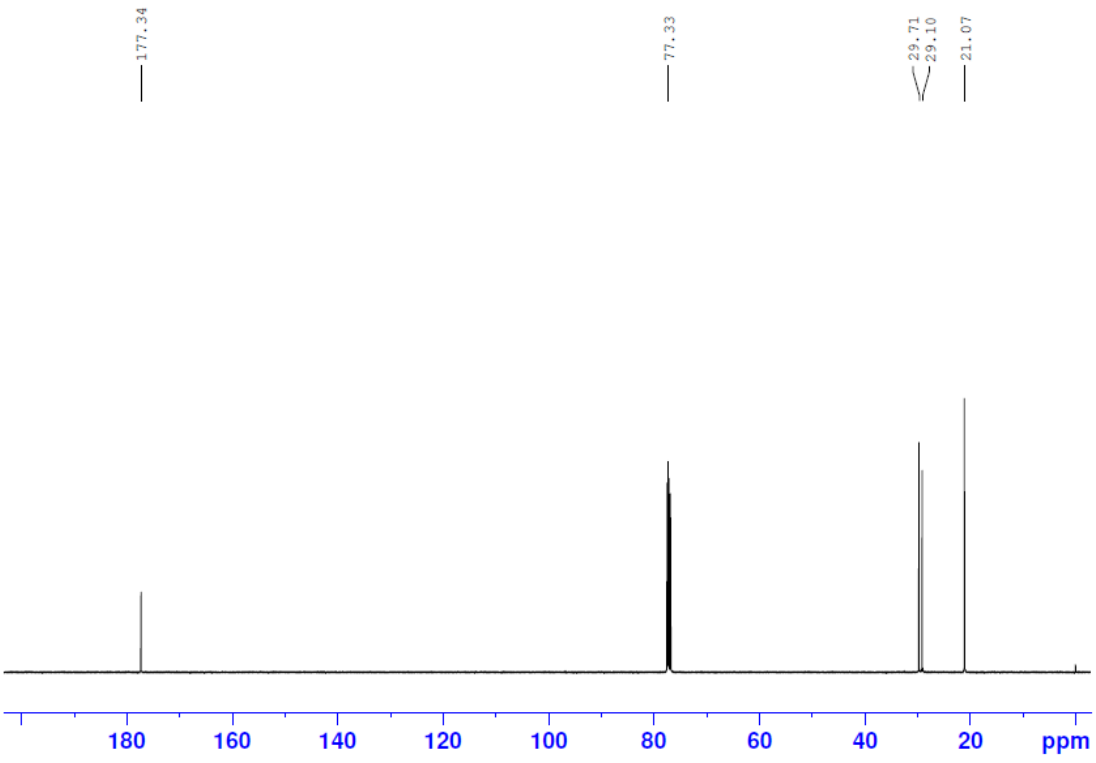

Figure 2.^{13}C NMR of GVL

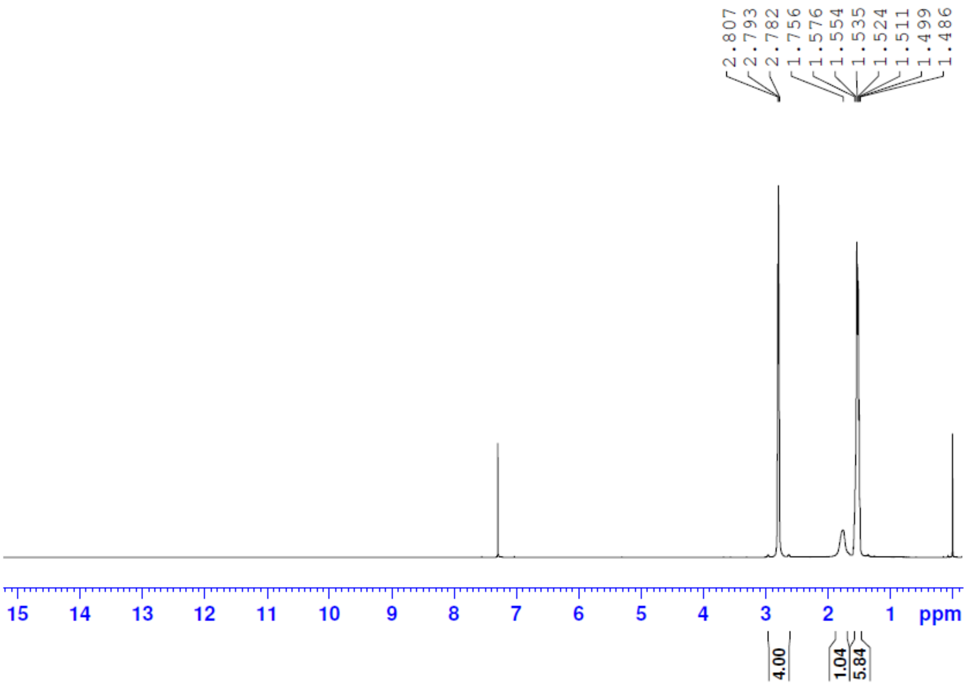

Figure 3.^{1}H NMR of PIP

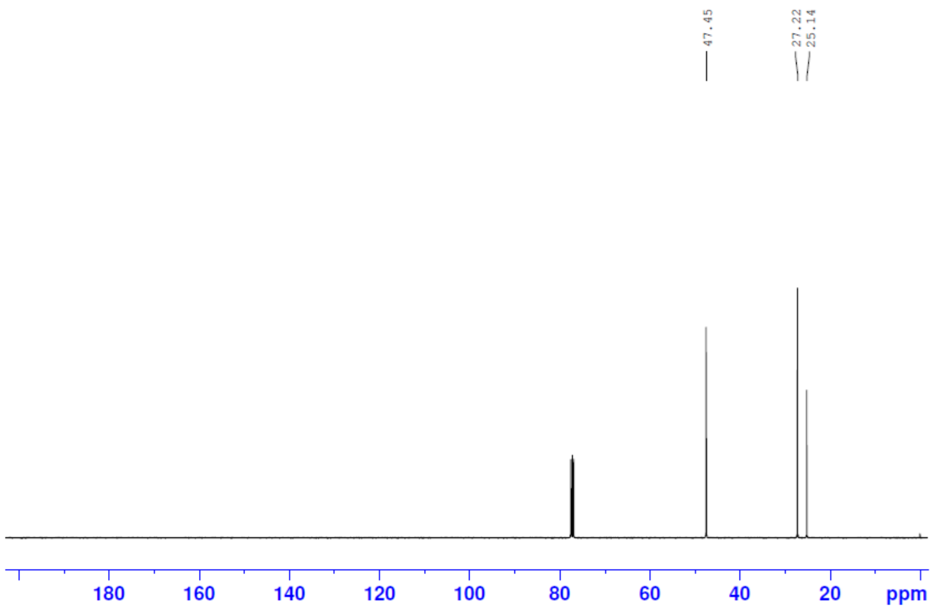

Figure 4.^{13}C NMR of PIP

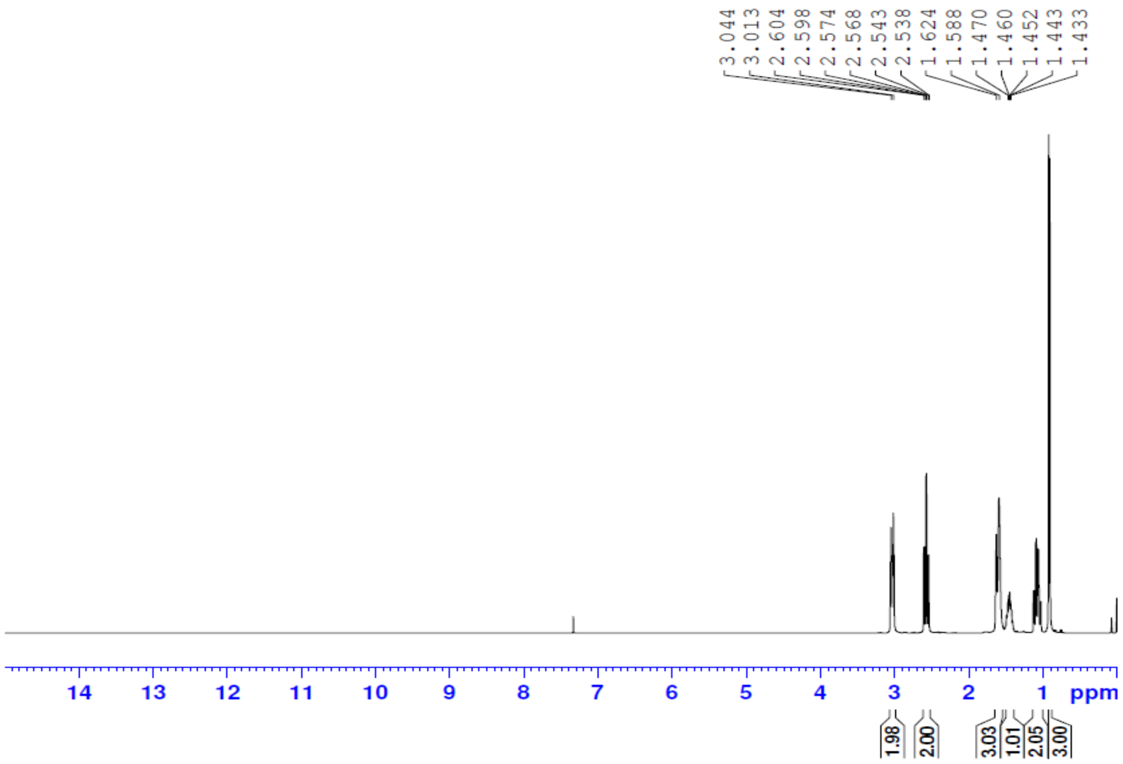

Figure 5.^{1}H NMR of 4-MP

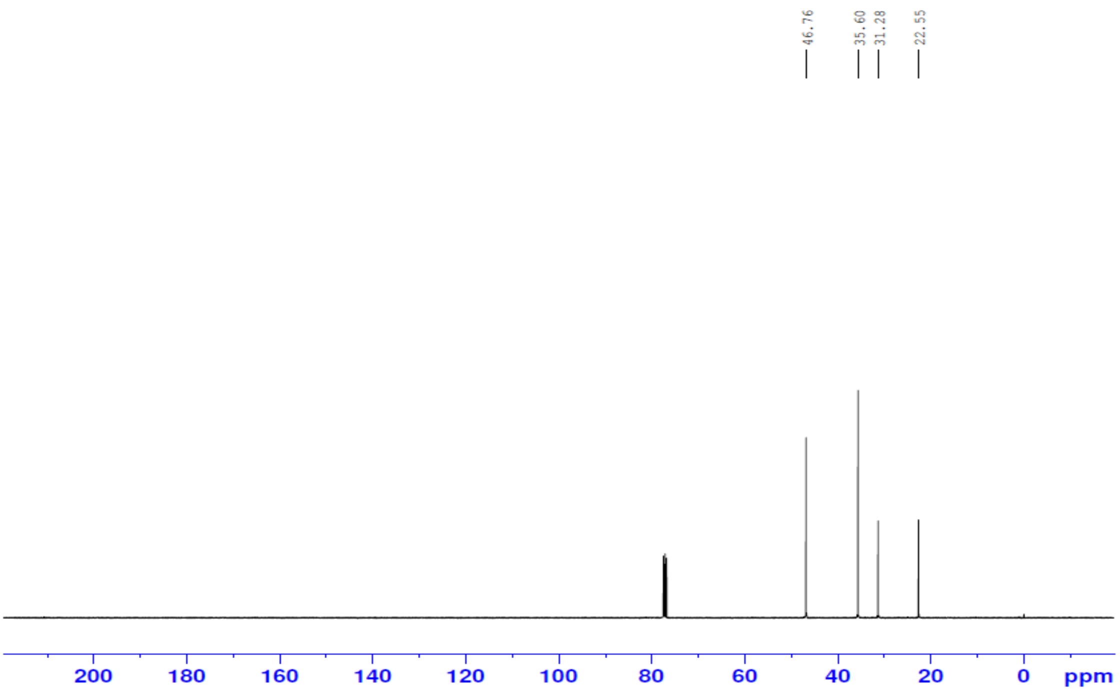

Figure 6.^{13}C NMR of 4-MP

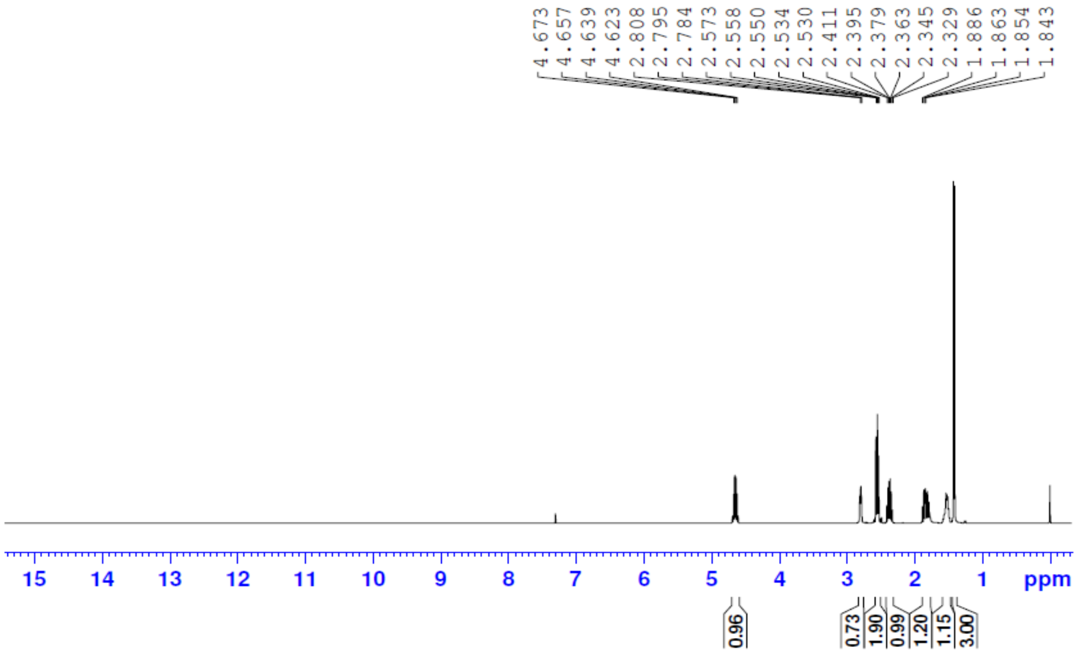

Figure 7.^{1}H NMR of 20% PIP in GVL at 0 min

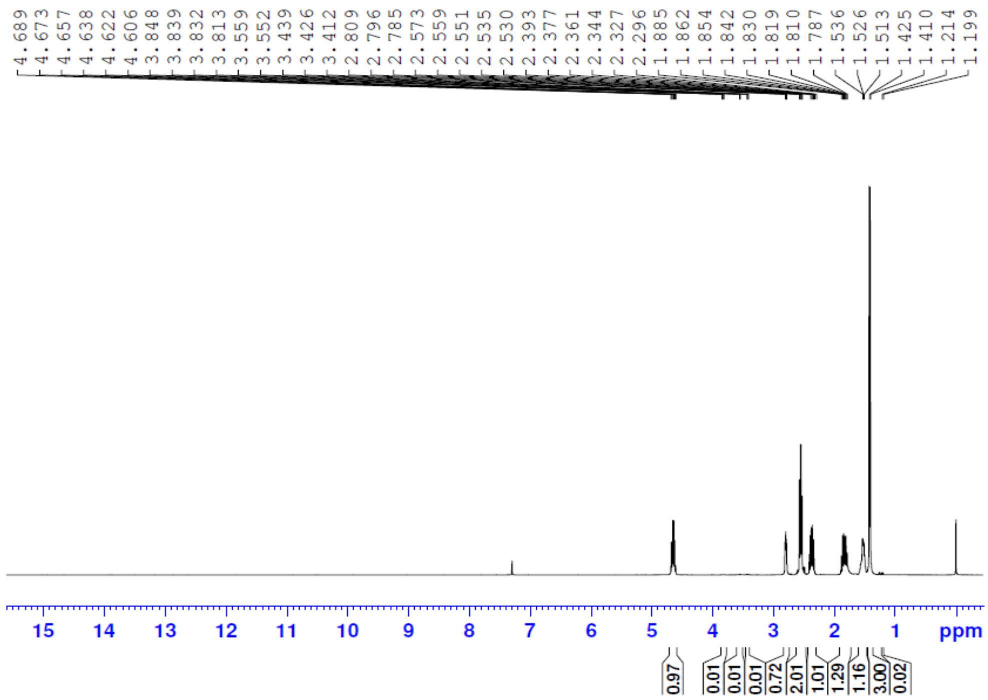

Figure 8.^{1}H NMR of 20% PIP in GVL after 2 h

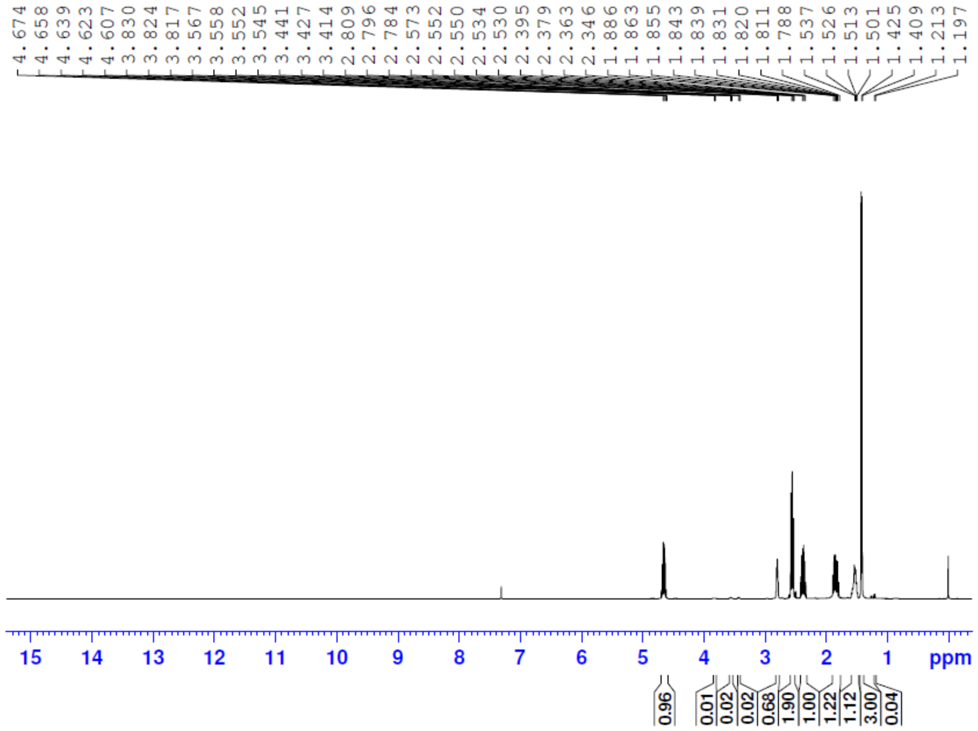

Figure 9.^{1}H NMR of 20% PIP in GVL after 4 h

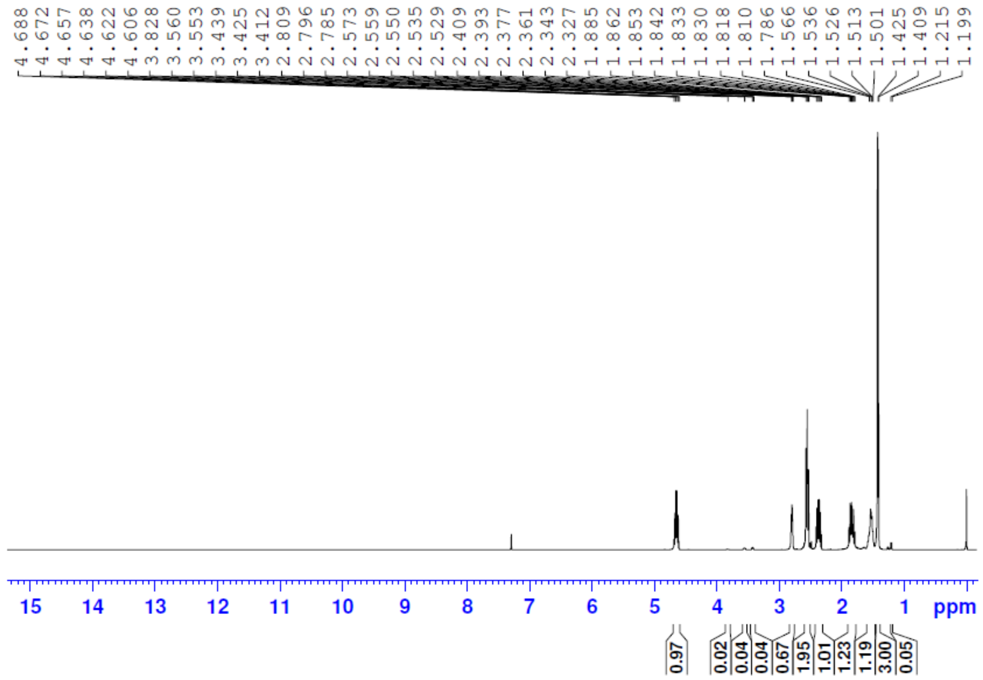

Figure 10.^{1}H NMR of 20% PIP in GVL after 6 h

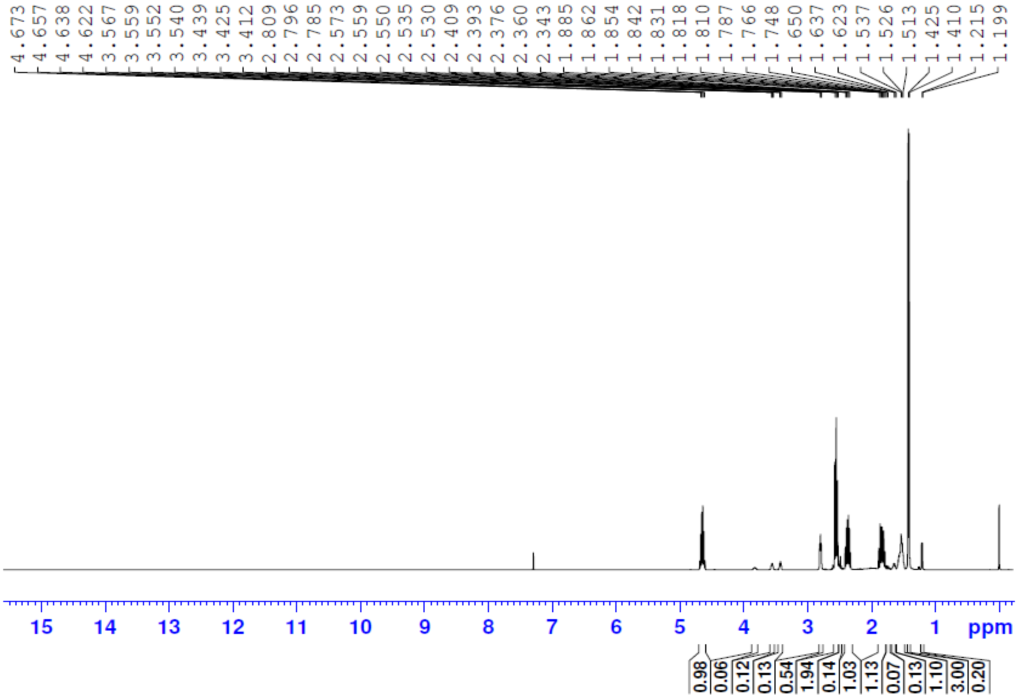

Figure 11.^{1}H NMR of 20% PIP in GVL after 24 h

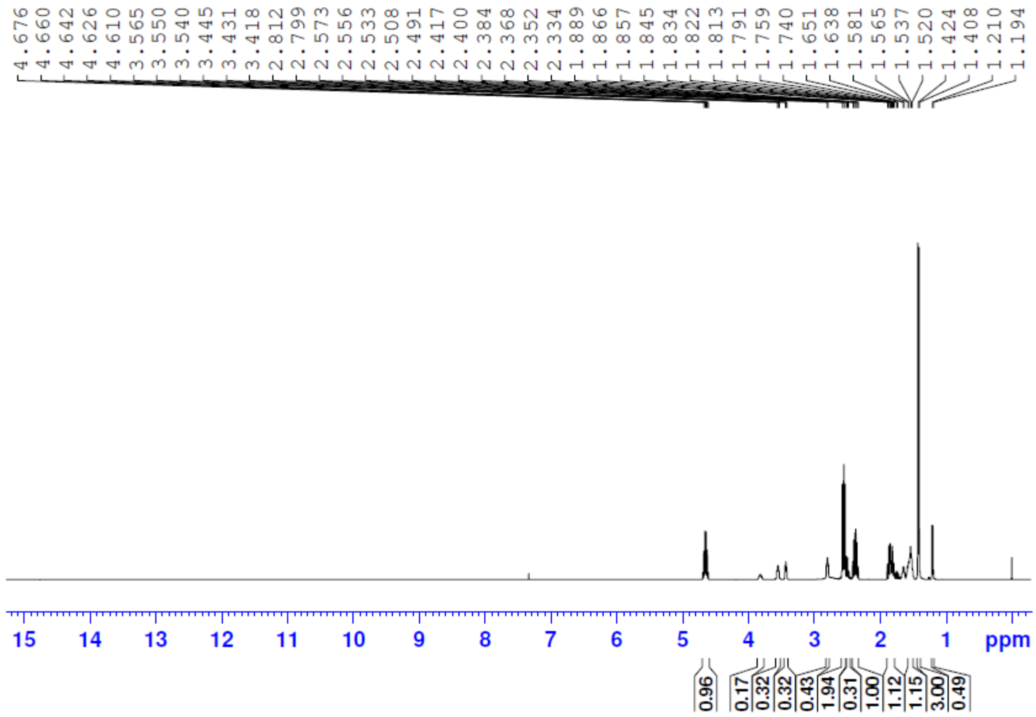

Figure 12. ^{1}H NMR of 20% PIP in GVL after 48 h

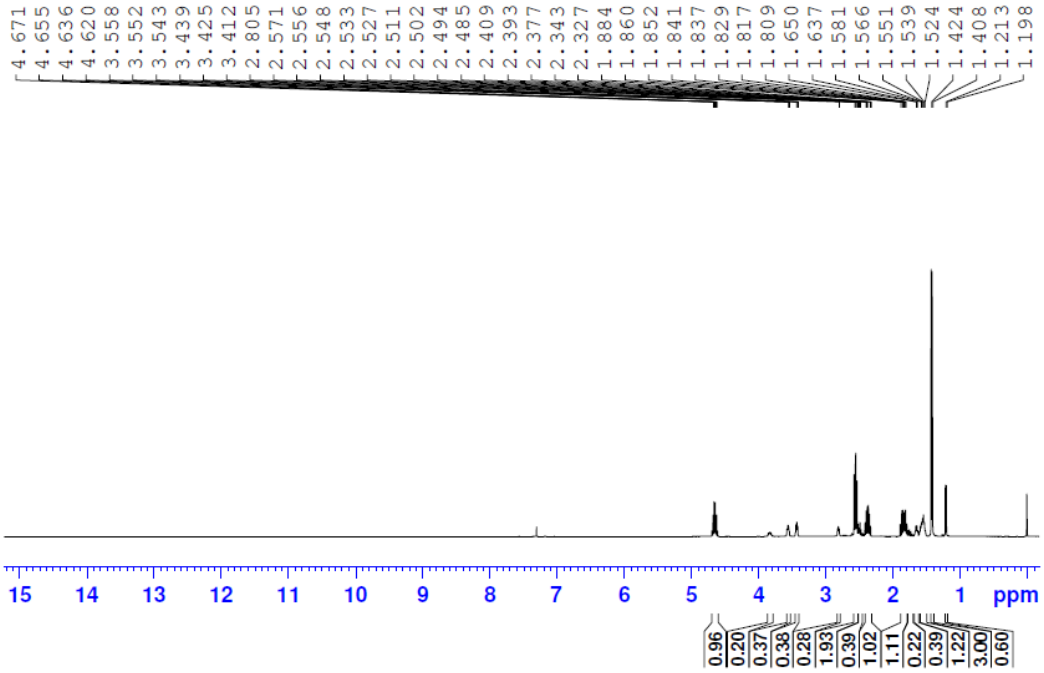

Figure 13. ^{1}H NMR of 20% PIP in GVL after 72 h

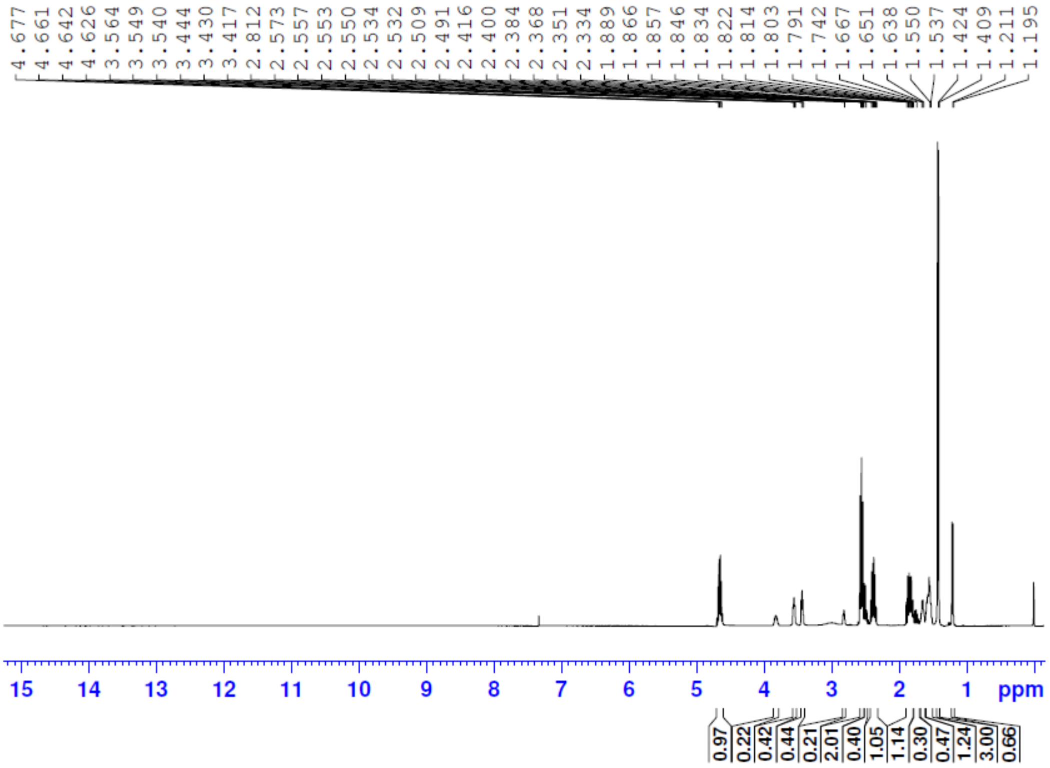

Figure 14.^{1}H NMR of 20% PIP in GVL after 96 h

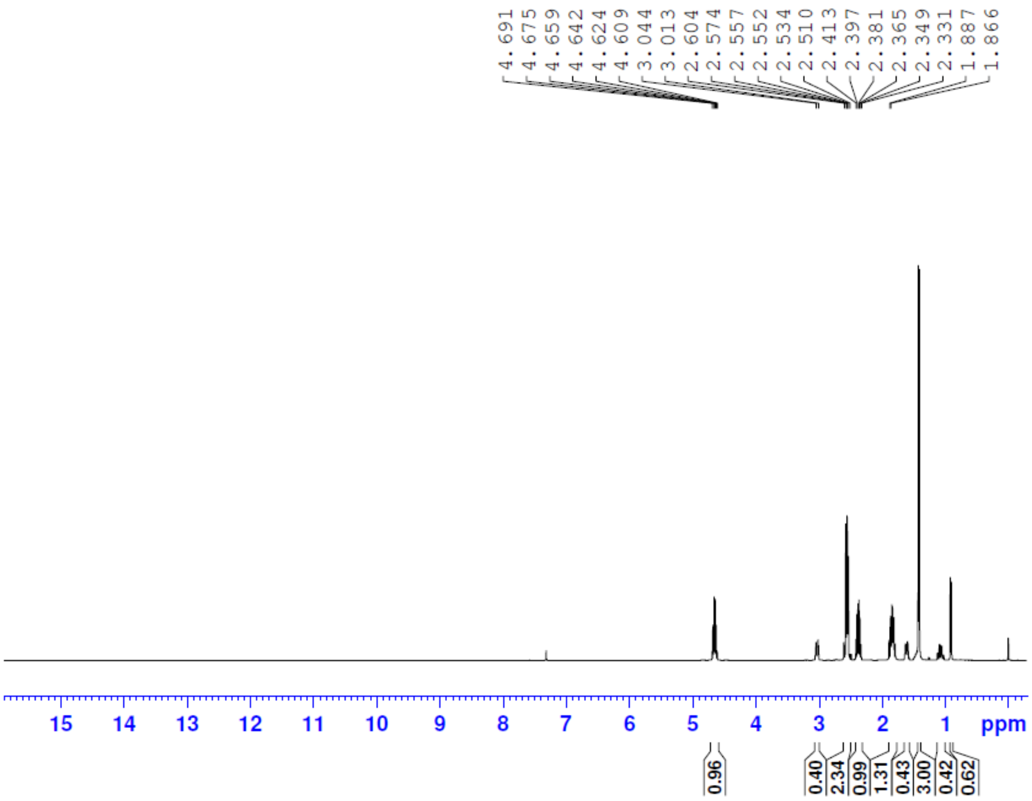

Figure 15.^{1}H NMR of 20% 4-MP in GVL at 0 min

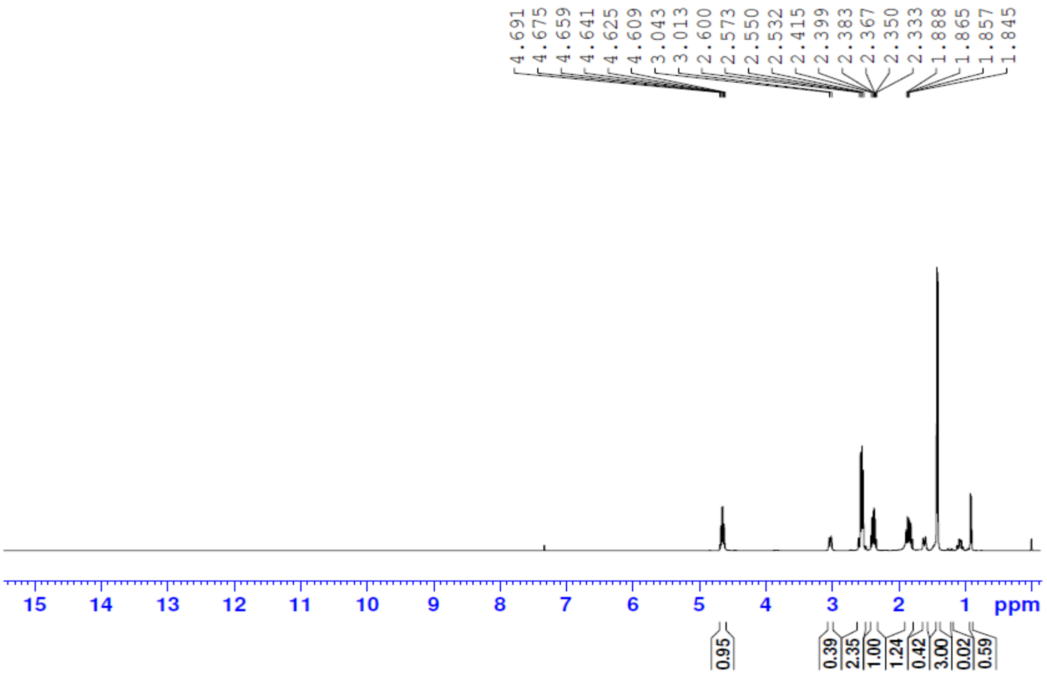

Figure 16.^{1}H NMR of 20% 4-MP in GVL after 2 h

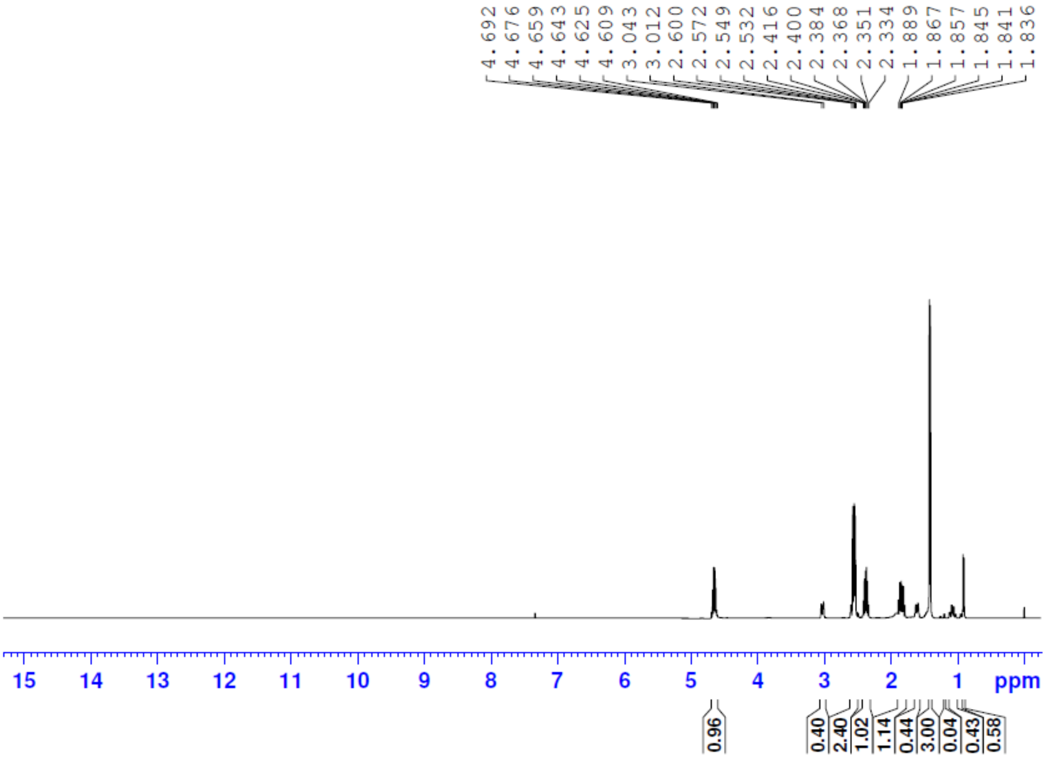

Figure 17.^{1}H NMR of 20% 4-MP in GVL after 4 h

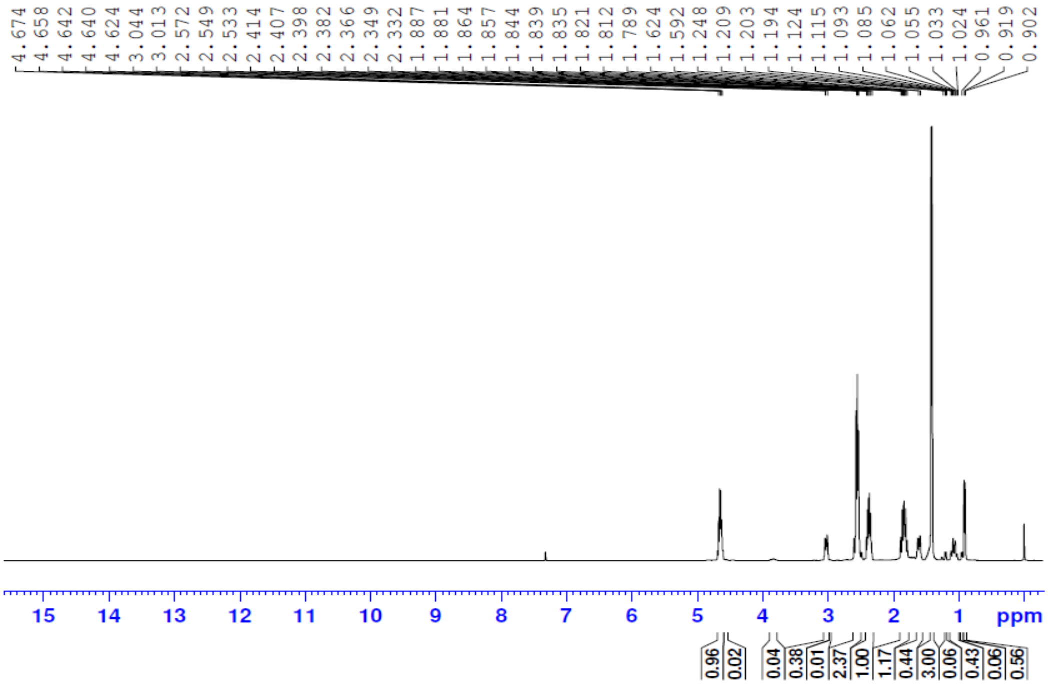

Figure 18.^{1}H NMR of 20% 4-MP in GVL after 6 h

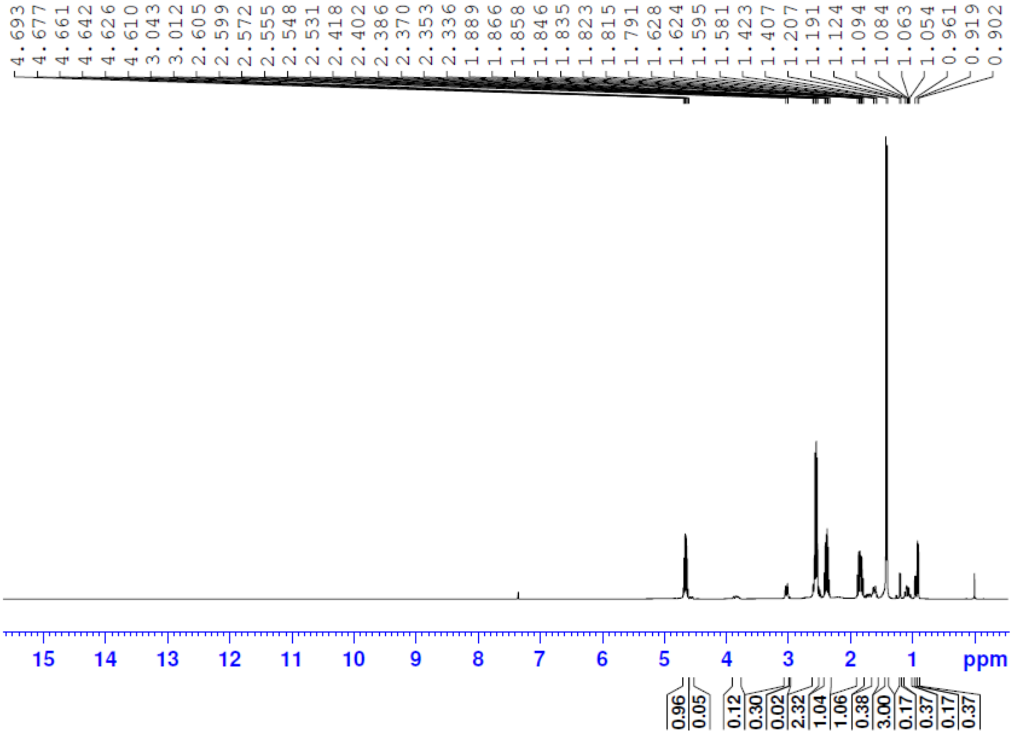

Figure 19.^{1}H NMR of 20% 4-MP in GVL after 24 h

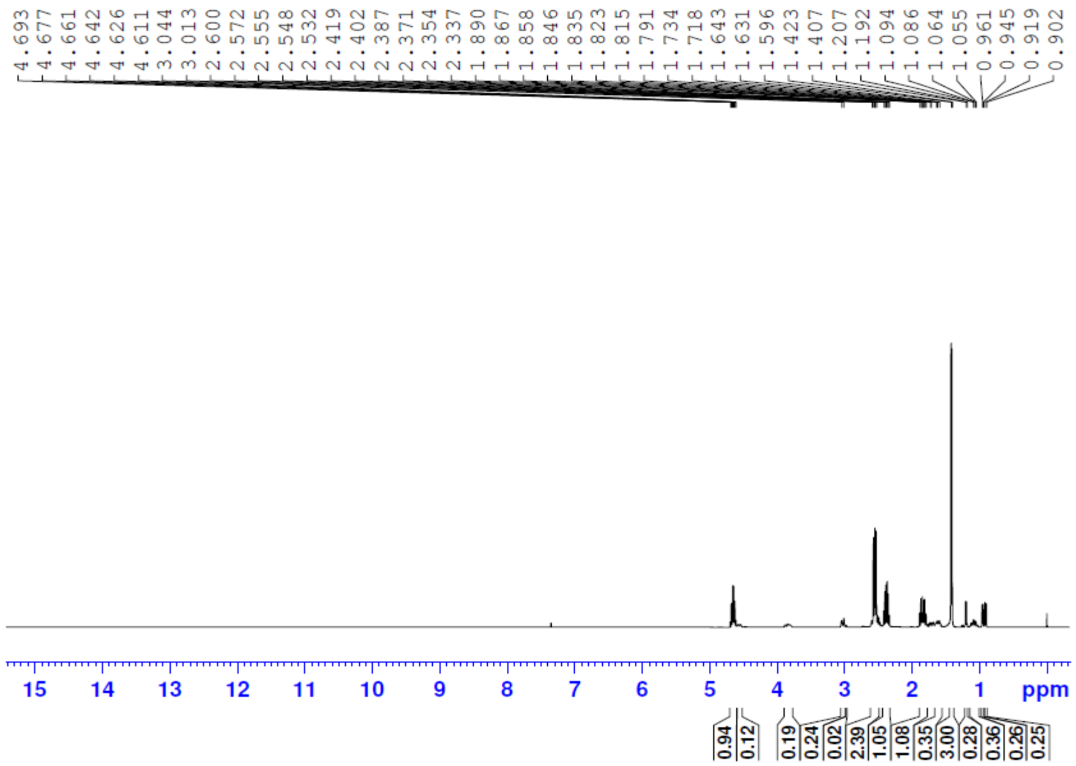

Figure 20.^{1}H NMR of 20% 4-MP in GVL after 48 h

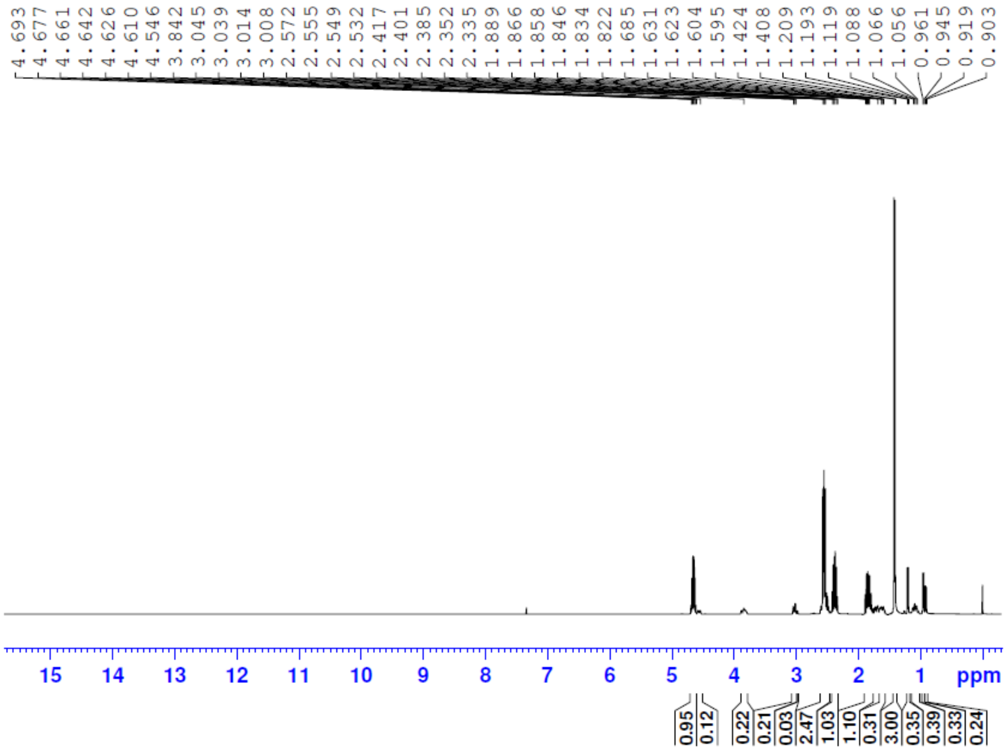

Figure 21.^{1}H NMR of 20% 4-MP in GVL after 72 h

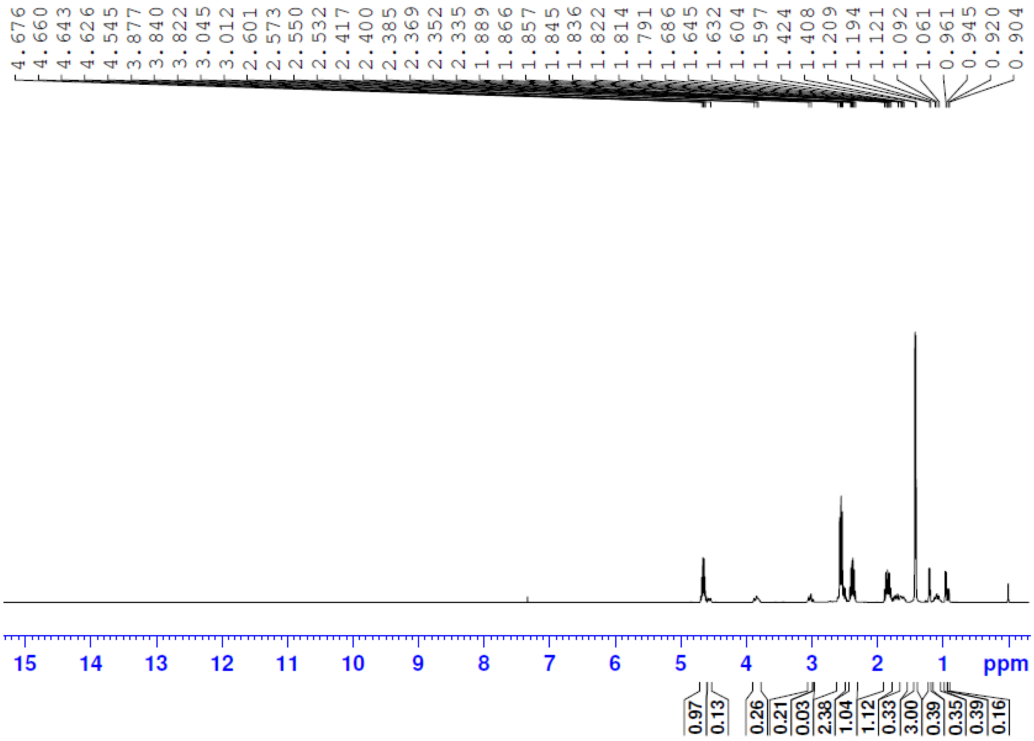

Figure 22.^{1}H NMR of 20% 4-MP in GVL after 96h

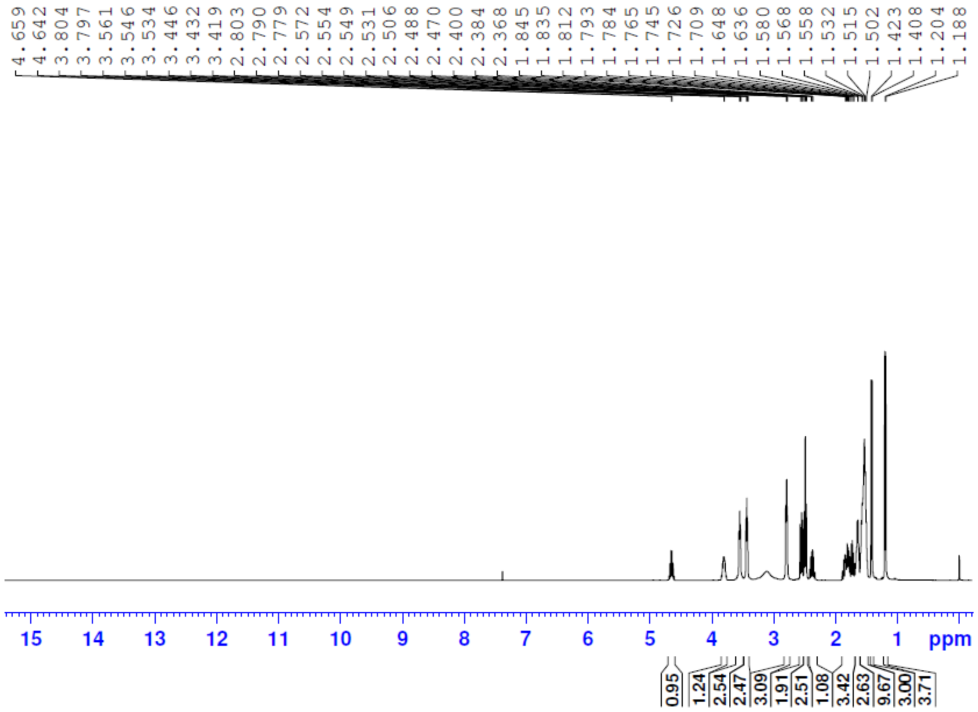

Figure 23.^{1}H NMR of equimolar solution of GVL and PIP after 1h MW at 90 ℃

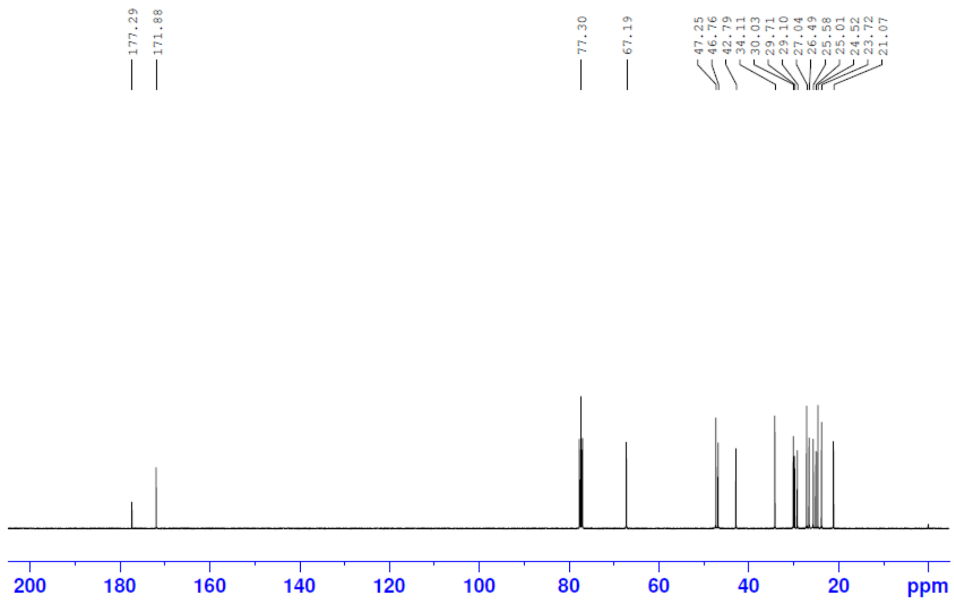

Figure 24.^{13}C NMR of equimolar solution of GVL and PIP after 1h MW at 90 ℃

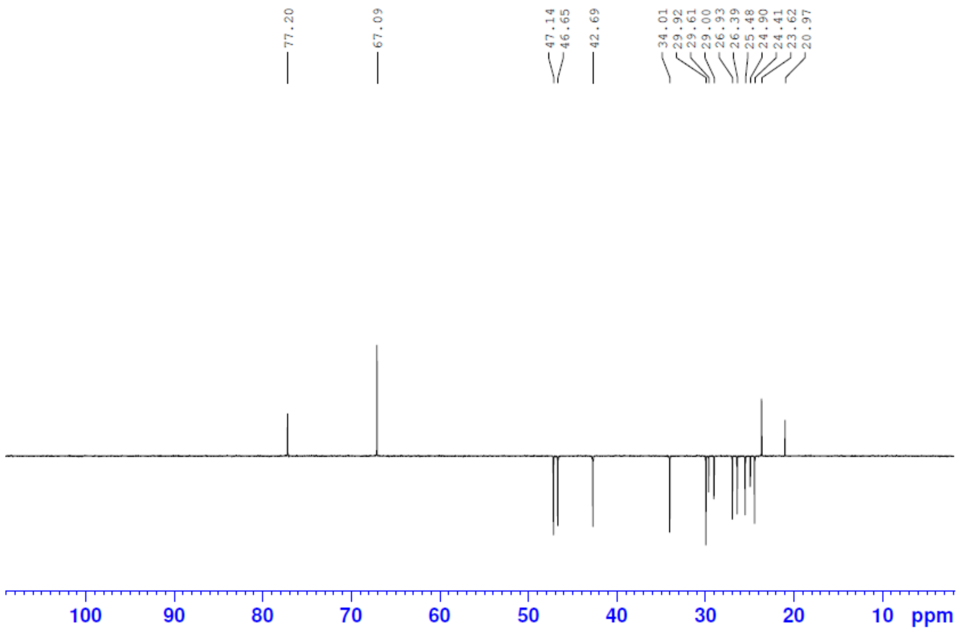

Figure 25. DEPT 135 NMR of equimolar solution of GVL and PIP after 1h MW at 90 ℃

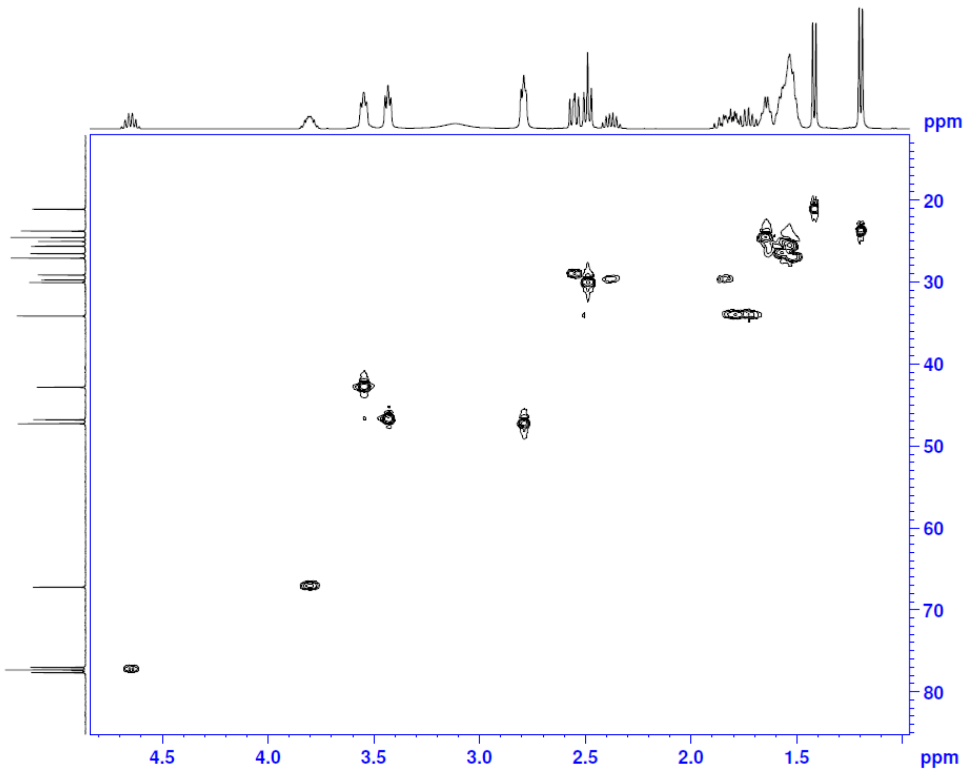

Figure 26. HSQC of equimolar solution of GVL and PIP after 1h MW at 90 ℃

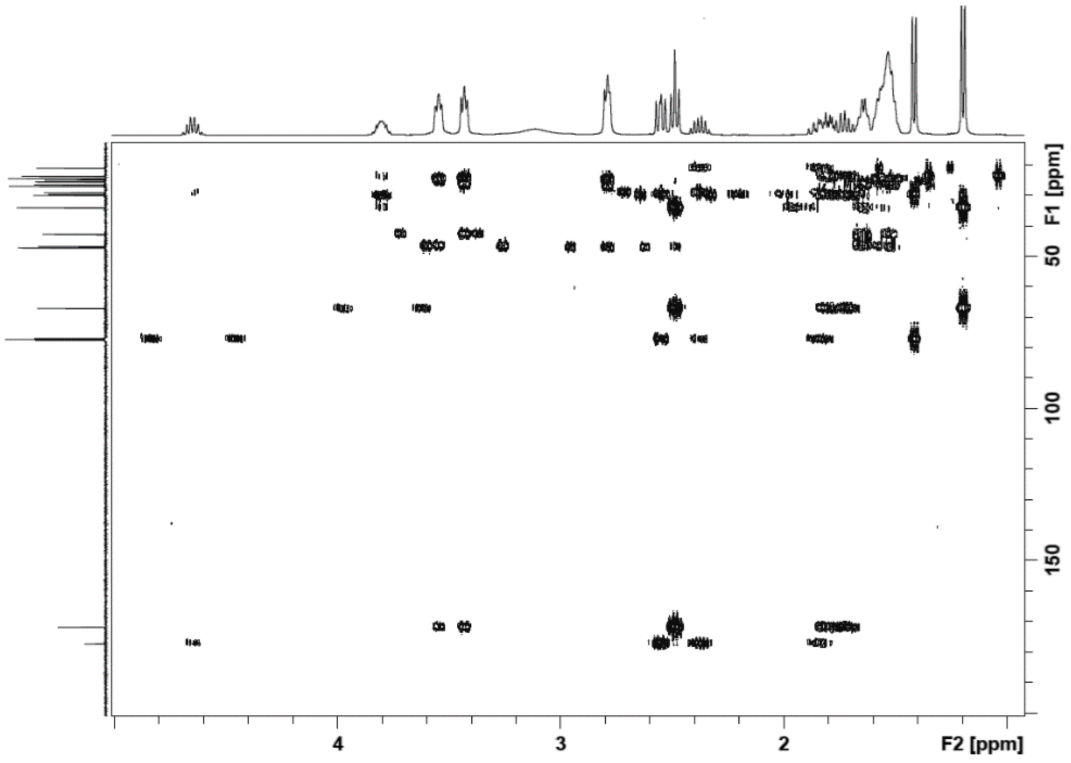

Figure 27. HMBC of equimolar solution of GVL and PIP after 1h MW at 90 ℃

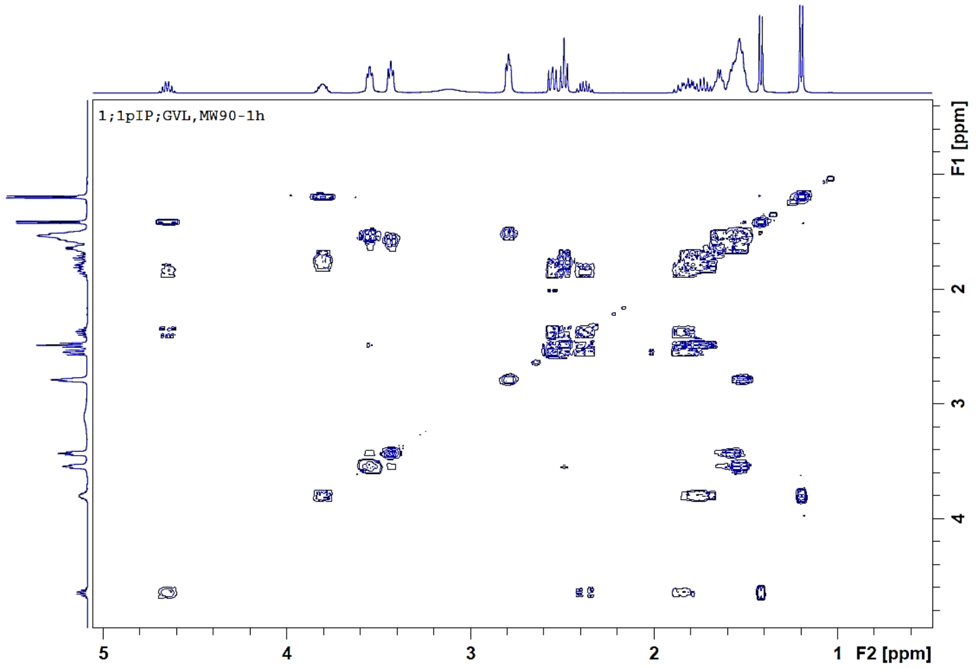

Figure 28. COSY of equimolar solution of GVL and PIP after 1h MW at 90 ℃

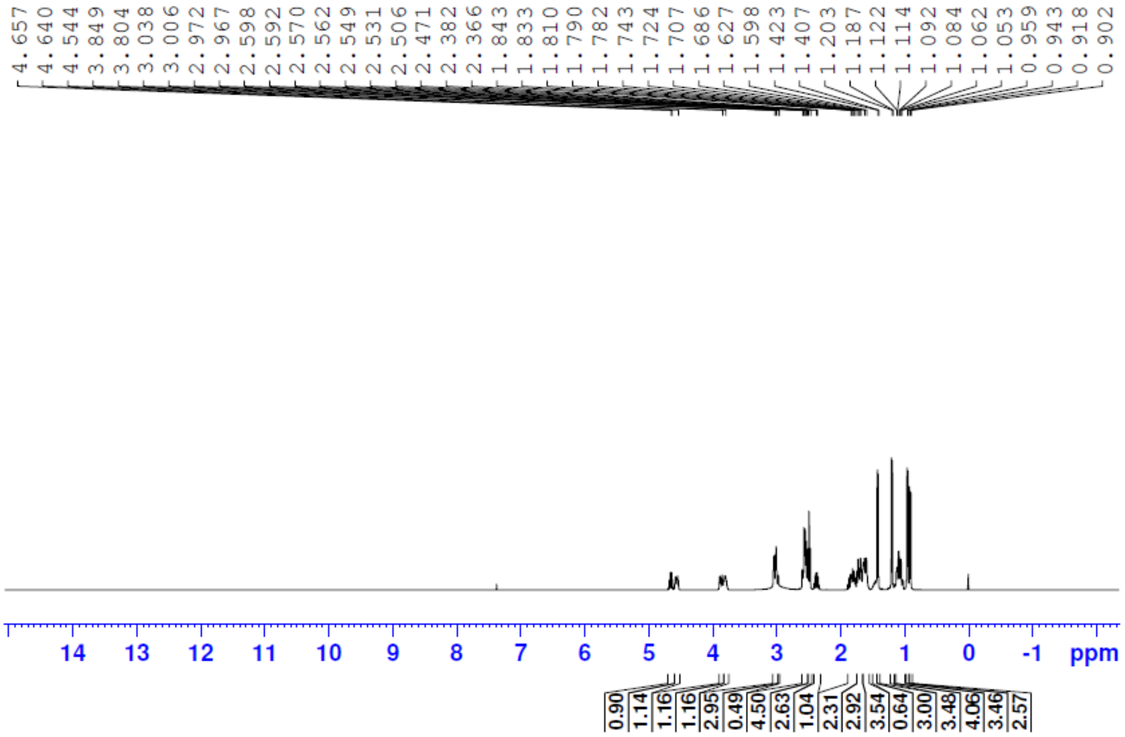

Figure 29. ^{1}H NMR of equimolar solution of GVL and 4-MP after 1h MW at 90 ℃

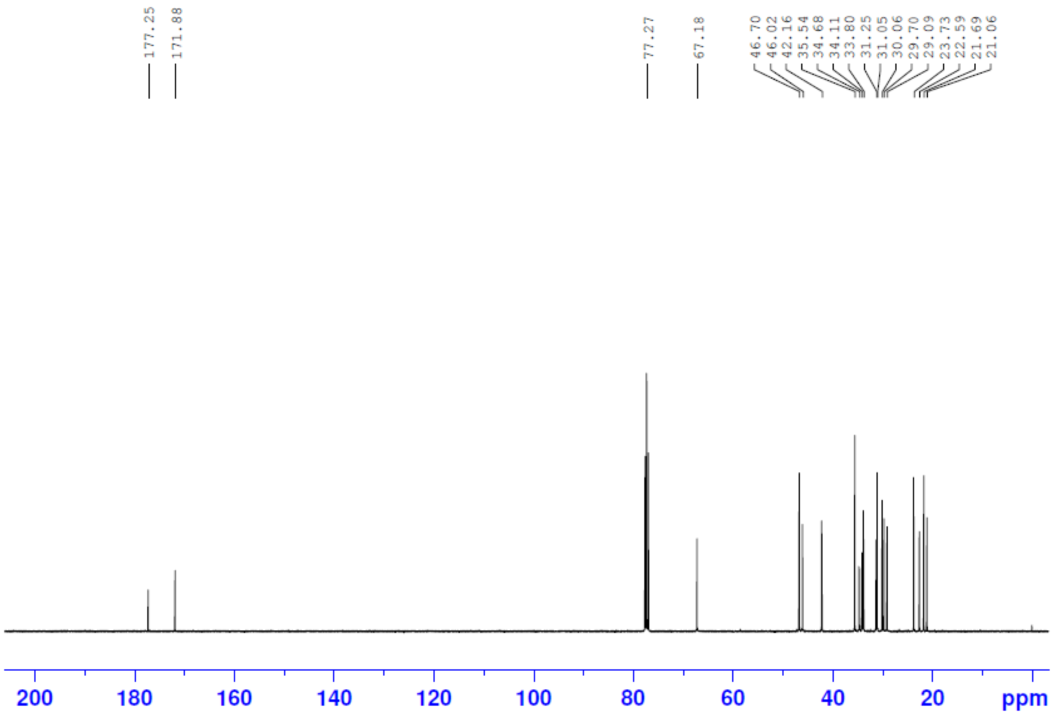

Figure 30. ^{13}C NMR of equimolar solution of GVL and 4-MP after 1h MW at 90 ℃

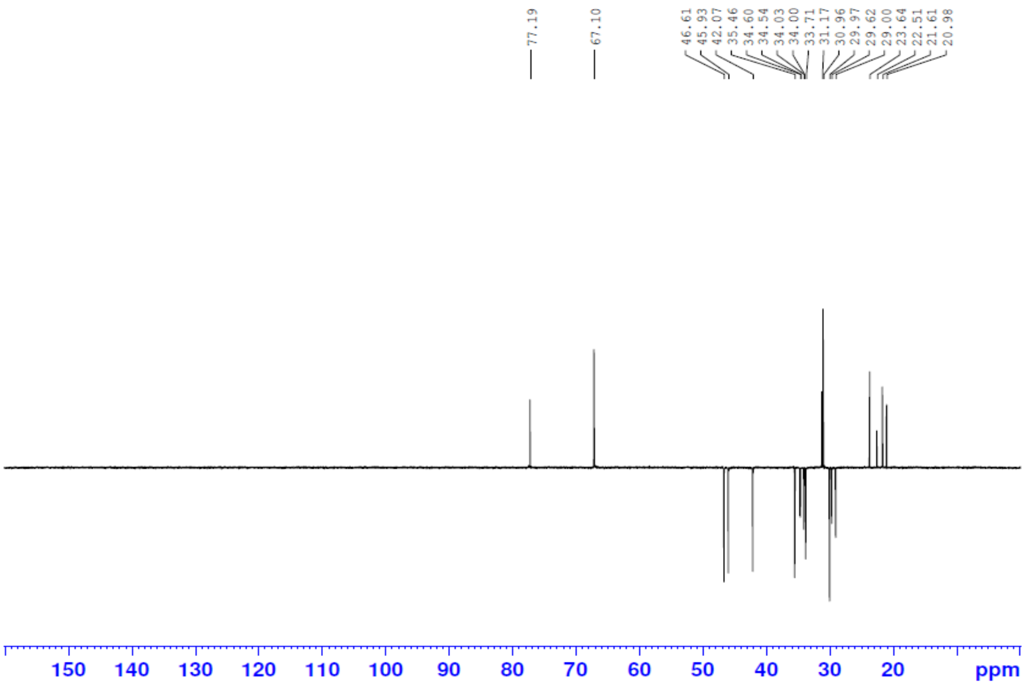

Figure 31. DEPT 135 of equimolar solution of GVL and 4-MP after 1h MW at 90 ℃

31. Table 1. ^{1}H (400MHz), ^{13}C (100MHz) NMR and HBMC correlation of mixture of Product (**3a)** GVL and PIP

S.N.	^{13}C	HSQC	HMBC
1	177.2, C		
2	171.8, C		
3	77.2, CH_2	4.64	177.2, 29.0
4	67.3, CH_2	3.80	30.0
5	47.3, CH_2	2.78	47.3, 25.1
6	46.7, CH_2	3.42	171.8, 42.8, 26.4, 24.5
7	42.8, CH_2	3.54	171.8, 46.7, 24.5
8	34.0, CH_2	1.80, 1.73	171.8, 67.3, 23.7
9	30.0, CH_2	2.48	171.8, 67.3, 34.0
10	29.7, CH_2	2.37, 1.83	177.2, 29.1, 21.0
11	29.0, CH_2	2.54	177.2, 77.3, 29.7
12	27.1, CH_2	1.51	47.3, 27.2
13	26.4, CH_2	1.56	46.7, 25.5
14	25.5, CH_2	1.53	42.8, 26.4
15	25.0, CH_2	1.54	47.3
16	24.5, CH_2	1.64	46.7, 42.8, 26.4, 25.5
17	23.7, CH_3	1.20	67.3, 34.0
18	21.0, CH_3	1.42	77.2, 29.7

32. Cartesian Coordinates for GVL

```
# opt freq b3lyp/6-311++g(d,p) geom=connectivity

Title Card Required

0 1
 C                  -0.46421691   -2.02938416    0.16968728
 C                   1.03212722   -1.77689887    0.32857778
 C                  -0.03308555    0.20830045    0.55725610
 C                  -0.86904336   -0.68113472   -0.36423812
 H                  -0.72143220   -2.84260435   -0.47638795
 H                  -0.90564501   -2.21791732    1.12598038
 H                   1.47580639   -1.60517197   -0.62983689
 H                  -1.92352816   -0.50271869   -0.33062974
 H                  -0.54567284   -0.55801271   -1.37674613
 O                  -0.26438007    1.42309043    0.79040567
 O                   1.07112529   -0.55847077    1.13135974
 C                   1.76866489   -2.94920086    1.00298145
 H                   1.67844658   -3.82457648    0.39431227
 H                   2.80290308   -2.70088169    1.11955309
 H                   1.33639520   -3.13666592    1.96365818

 1 2 1.0 4 1.0 5 1.0 6 1.0
 2 7 1.0 11 1.0 12 1.0
 3 4 1.0 10 2.0 11 1.0
 4 8 1.0 9 1.0
 5
 6
 7
 8
 9
 10
 11
 12 13 1.0 14 1.0 15 1.0
 13
 14
 15
```

33. Coordinates for PIP

```
# opt freq b3lyp/6-311++g(d,p) geom=connectivity

Title Card Required

0 1
 C                  -2.67620952   -2.84380601    0.04207373
 C                  -1.12628081   -2.88283691   -0.03726108
 C                  -0.56947933   -1.43538667   -0.10783526
 C                  -1.46586451   -0.58547088    0.73225457
 C                  -3.12274320   -1.68343399   -0.78602352
 H                   0.44888390   -1.44294393    0.22046219
 H                  -0.79496123   -3.40780391    0.83425311
 H                  -0.77289576   -3.38270760   -0.91484201
 H                  -2.61672628   -1.73768905   -1.72724786
 H                  -3.01700505   -2.65852499    1.03928451
 H                  -1.60411163   -1.08132762    1.67029179
```

```
 H                  -1.06280140    0.39016604    0.90710517
 H                  -4.17927336   -1.68092073   -0.95525029
 H                  -3.06083158   -3.78476272   -0.29194174
 H                  -0.61575042   -1.04149819   -1.10162139
 N                  -2.75750696   -0.44130670   -0.02264446
 H                  -2.70323162    0.28353416   -0.70941947

 1 2 1.0 5 1.0 10 1.0 14 1.0
 2 3 1.0 7 1.0 8 1.0
 3 4 1.0 6 1.0 15 1.0
 4 11 1.0 12 1.0 16 1.0
 5 9 1.0 13 1.0 16 1.0
 6
 7
 8
 9
 10
 11
 12
 13
 14
 15
 16 17 1.0
 17
```

34. Coordinates of 4-MP

```
# opt freq b3lyp/6-311++g(d,p) geom=connectivity

Title Card Required

0 1
 C                  -2.67620952   -2.84380601    0.04207373
 C                  -1.12628081   -2.88283691   -0.03726108
 C                  -0.56947933   -1.43538667   -0.10783526
 C                  -1.46586451   -0.58547088    0.73225457
 C                  -3.12274320   -1.68343399   -0.78602352
 H                   0.44888390   -1.44294393    0.22046219
 H                  -0.79496123   -3.40780391    0.83425311
 H                  -2.61672628   -1.73768905   -1.72724786
 H                  -3.01700505   -2.65852499    1.03928451
 H                  -1.60411163   -1.08132762    1.67029179
 H                  -1.06280140    0.39016604    0.90710517
 H                  -4.17927336   -1.68092073   -0.95525029
 H                  -3.06083158   -3.78476272   -0.29194174
 H                  -0.61575042   -1.04149819   -1.10162139
 N                  -2.75750696   -0.44130670   -0.02264446
 H                  -2.70323162    0.28353416   -0.70941947
 C                  -0.61767055   -3.60227697   -1.30032149
 H                   0.43902485   -3.75229351   -1.22423023
 H                  -1.10666592   -4.54952456   -1.39254699
 H                  -0.83198569   -3.00488329   -2.16176784

 1 2 1.0 5 1.0 9 1.0 13 1.0
 2 3 1.0 7 1.0 17 1.0
 3 4 1.0 6 1.0 14 1.0
 4 10 1.0 11 1.0 15 1.0
```

```
 5 8 1.0 12 1.0 15 1.0
 6
 7
 8
 9
 10
 11
 12
 13
 14
 15 16 1.0
 16
 17 18 1.0 19 1.0 20 1.0
 18
 19
 20
```

35. Coordinates for TS for GVL+PIP

```
# opt=(calcfc,ts) freq b3lyp/6-31++g(d,p) geom=connectivity

Title Card Required

0 1
 C                  2.79608000   -0.82937400   -0.32050900
 C                  1.44727700   -1.56441900   -0.33516800
 C                  0.53133000   -0.89183200    0.67448300
 C                  2.48434900    0.64629800   -0.66384300
 H                  3.23037700   -0.89631700    0.68468400
 H                  3.50016100   -1.28877700   -1.02539800
 H                  1.54896100   -2.61330200   -0.03679900
 H                  1.03362800   -1.53058200   -1.34890900
 H                  2.35779300    0.70605100   -1.76738200
 C                 -1.49493800    0.53204000    1.14975700
 C                 -2.62791700    1.36409100    0.54767600
 C                 -3.57592700    0.50352900   -0.29943100
 C                 -2.78373800   -0.26835700   -1.36291700
 C                 -1.64245200   -1.07361700   -0.73496100
 H                 -3.17029500    1.85092200    1.36590200
 H                 -1.86864700   -0.21329100    1.85862200
 H                 -0.76080500    1.15124100    1.66503400
 H                 -4.10612600   -0.20767900    0.34933100
 H                 -4.33994600    1.12811500   -0.77507300
 H                 -3.43263600   -0.95771400   -1.91513200
 H                 -2.36547900    0.43282500   -2.09737900
 H                 -2.03430500   -1.85136900   -0.06736400
 H                 -1.04082500   -1.55949400   -1.50479300
 H                 -2.20028700    2.16602400   -0.06850600
 O                  1.29437100    0.97910500   -0.01299900
 O                  0.54479200   -1.10103100    1.86622200
 N                 -0.75132600   -0.19761000    0.07707000
 H                 -0.12781400    0.52763800   -0.43940200
 C                  3.63020500    1.58704000   -0.27556700
 H                  4.56779600    1.30914000   -0.77518200
 H                  3.38266000    2.61647000   -0.55337100
 H                  3.78616700    1.55638500    0.80872000
```

```
 1 2 1.0 4 1.0 5 1.0 6 1.0
 2 3 1.0 7 1.0 8 1.0
 3 26 2.0
 4 9 1.0 25 1.0 29 1.0
 5
 6
 7
 8
 9
 10 11 1.0 16 1.0 17 1.0 27 1.0
 11 12 1.0 15 1.0 24 1.0
 12 13 1.0 18 1.0 19 1.0
 13 14 1.0 20 1.0 21 1.0
 14 22 1.0 23 1.0 27 1.0
 15
 16
 17
 18
 19
 20
 21
 22
 23
 24
 25
 26
 27
 28
 29 30 1.0 31 1.0 32 1.0
 30
 31
 32
```

36. Coordinates for TS for GVL+PIP

```
# opt=(calcfc,ts) freq b3lyp/6-31++g(d,p) geom=connectivity

Title Card Required

0 1
 C                  3.20123400   -0.55953500   -0.54521600
 C                  1.92665300   -1.41165300   -0.64044400
 C                  1.01366600   -1.02797200    0.51301800
 C                  2.73681400    0.91495800   -0.59630400
 H                  3.69769900   -0.76299100    0.41175600
 H                  3.90204400   -0.81117600   -1.35091000
 H                  2.14156800   -2.48136900   -0.54530700
 H                  1.45150500   -1.23855800   -1.61237200
 H                  2.54061800    1.15951900   -1.66352800
 C                 -1.09904700    0.08299400    1.32379600
 C                 -2.33252900    0.89417900    0.92976700
 C                 -3.26888300    0.11956100   -0.01309700
 C                 -2.46268600   -0.37775200   -1.22486400
 C                 -1.21920900   -1.16824900   -0.80888000
 H                 -2.86379100    1.17921100    1.84567800
 H                 -1.36336000   -0.80806800    1.90137000
 H                 -0.39430500    0.67061300    1.91194000
```

```
H                 -3.63642700   -0.76525700    0.53005400
H                 -3.08212800   -1.01708200   -1.86558200
H                 -2.15543200    0.48155600   -1.83751400
H                 -1.49827800   -2.08751200   -0.27889600
H                 -0.62752300   -1.44803900   -1.68215900
H                 -2.01509800    1.82918900    0.44730300
O                  1.56152900    1.00348700    0.15275500
O                  1.11532800   -1.44658200    1.64379700
N                 -0.35863000   -0.36930000    0.10685800
H                  0.16439800    0.49453700   -0.29426100
C                  3.81232800    1.88103100   -0.08737900
H                  4.74110600    1.79383500   -0.66677200
H                  3.45556700    2.91333400   -0.16001500
H                  4.03279200    1.67176800    0.96549000
C                 -4.47920300    0.95749800   -0.43899100
H                 -5.14743600    0.38782500   -1.09436300
H                 -5.05996400    1.28188200    0.43139000
H                 -4.16282500    1.85628500   -0.98215200

1 2 1.0 4 1.0 5 1.0 6 1.0
2 3 1.0 7 1.0 8 1.0
3 25 2.0
4 9 1.0 24 1.0 28 1.0
5
6
7
8
9
10 11 1.0 16 1.0 17 1.0 26 1.0
11 12 1.0 15 1.0 23 1.0
12 13 1.0 18 1.0 32 1.0
13 14 1.0 19 1.0 20 1.0
14 21 1.0 22 1.0 26 1.0
15
16
17
18
19
20
21
22
23
24
25
26
27
28 29 1.0 30 1.0 31 1.0
29
30
31
32 33 1.0 34 1.0 35 1.0
33
34
35
```

Figure 31: Optimized geometry of GVL, PIP and 4-MP with bond lengths

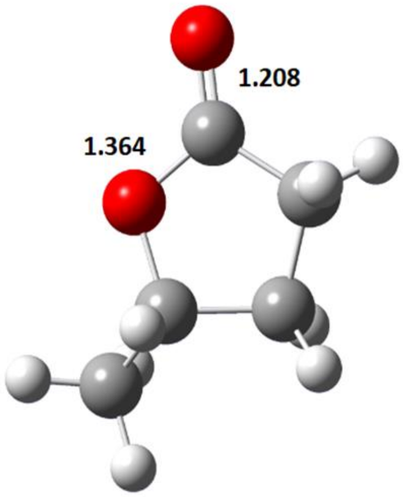

GVL

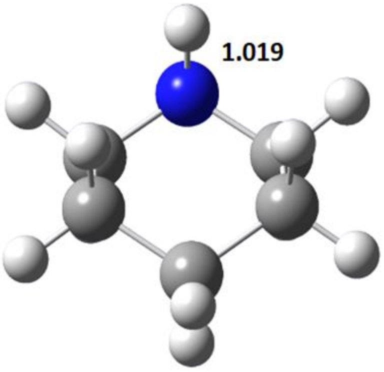

PIP

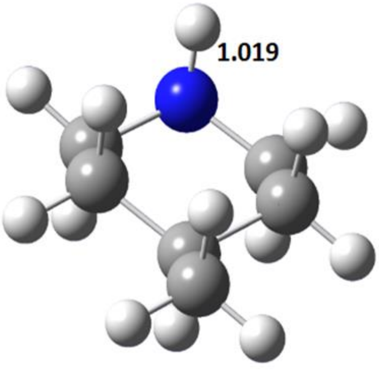

4-MP
